# Environmental metagenome classification for constructing a microbiome fingerprint

**DOI:** 10.1186/s13062-019-0251-z

**Published:** 2019-11-13

**Authors:** Jolanta Kawulok, Michal Kawulok, Sebastian Deorowicz

**Affiliations:** Institute of Informatics, Silesian University of Technology, Gliwice, Poland

**Keywords:** Metagenome, MetaSUB, Urban microbiome, Environmental classification, Sequence classification, CAMDA challenge, K-mers

## Abstract

**Background:**

Nowadays, not only are single genomes commonly analyzed, but also metagenomes, which are sets of, DNA fragments (reads) derived from microbes living in a given environment. Metagenome analysis is aimed at extracting crucial information on the organisms that have left their traces in an investigated environmental sample.In this study we focus on the MetaSUB Forensics Challenge (organized within the CAMDA 2018 conference) which consists in predicting the geographical origin of metagenomic samples. Contrary to the existing methods for environmental classification that are based on taxonomic or functional classification, we rely on the similarity between a sample and the reference database computed at a reads level.

**Results:**

We report the results of our extensive experimental study to investigate the behavior of our method and its sensitivity to different parameters. In our tests, we have followed the protocol of the MetaSUB Challenge, which allowed us to compare the obtained results with the solutions based on taxonomic and functional classification.

**Conclusions:**

The results reported in the paper indicate that our method is competitive with those based on taxonomic classification. Importantly, by measuring the similarity at the reads level, we avoid the necessity of using large databases with annotated gene sequences. Hence our main finding is that environmental classification of metagenomic data can be proceeded without using large databases required for taxonomic or functional classification.

**Reviewers:**

This article was reviewed by Eran Elhaik, Alexandra Bettina Graf, Chengsheng Zhu, and Andre Kahles.

## Background

Recently, we may witness rapid development of nucleotide sequencing. Not only are single genomes commonly analyzed, but also *metagenomes*, which are sets of DNA fragments (reads) derived from microbes living in a given environment [1]. Microbiome is a complex community of bacteria, fungi, viruses, and micro-eukaryotes. Metagenome analysis is therefore aimed at extracting different kinds of information on the organisms that have left their traces in an investigated environmental sample. As a result, it helps in creating a general profile of the place that the samples were extracted from.

Metagenomic data analysis may consist in *supervised* and/or *unsupervised* classification (the latter is commonly referred to as *clustering*) of the metagenomic reads. During the supervised classification, the reads from a presented sample are compared against a database containing groups of reference sequences. Depending on the main goal of the study, the metagenomic data can be subject to three main types of supervised classification, namely: (*i*) *taxonomic classification*—to identify the organisms in the sample; (*ii*) *functional classification*—to determine the functions that can be performed by the microorganisms from the sample; and (*iii*) *environmental classification*—to identify the origin of the sample. The metagenomic data may also be subject to clustering (i.e., *binning*). However, it is usually performed as a preprocessing step that precedes further analysis with the use of reference databases. A metagenome is a mixture of fragments from different genomes, hence it is attempted in some studies to recover each individual genome. First, the metagenome reads are assembled into contigs, and later the binning is performed to group them into genomes [2–4].

Metagenome classification is an active research topic, and there are many studies which explore the aforementioned classification scenarios [5]. Huson et al. introduced the MEGAN-LR program [6] which compares long reads against the NCBI-nr protein reference database. In this way, they directly perform both taxonomic and functional classification, which subsequently allows them to identify the origin of an investigated sample. In the MetaBinG2 program [7], Qiao et al. decompose the complete genome sequence into short substrings composed of *k* symbols (*k*-mers), and then a Markov model for their probability vector is created to perform taxonomic classification. Based on the organisms identified in different samples, the similarity between the latter is computed, which makes it possible to classify an investigated sample to the most probable environment. Some other attempts make use of the spaced *k*-mers [8] or the 16S gene profile for the analysis [9–12]. In particular, Walker et al. [9] used that for taxonomic classification prior to the environmental classification. Moreover, some programs are employed for metagenomic strain identification (e.g., MetaMLST [13], StrainPhlAn [14], PanPhlAn [15]), which helps to analyze the metagenome samples [16–19]. Recently, Gerner et al. developed a method for in silico creation of artificial communities that can be used as a gold standard for validating various metagenome approaches [20].

In this paper, we report our research aimed at approaching the MetaSUB Forensics Challenge, which was organized within the CAMDA 2018 competition (a track of the ISMB 2018 conference). Other solutions submitted for this competition are based on information extracted from the taxonomic and/or functional profiles of microbiota compositions. Ryan [21] performs taxonomic classification against the NCBI-nr database, followed by t-Distributed Stochastic Neighbor Embedding to reduce the dimensionality. Finally, the obtained feature vectors are classified using random forests. Walker and Datta [22], as well as Chierici et al. [23], proposed to exploit information on the taxonomic rank to extract the features that are later classified using random forests. Sanchez et al. [24] uses decision trees to classify the functional profiles created from the metagenomic data, whilst Zhu et al. employs support vector machines for classification [25].

Our contribution consists in testing the reliability of the microbiome fingerprints for identifying the sample origin directly from the metagenomic data—we exploited the data published within the MetaSUB Forensics Challenge. We demonstrate that it is not necessary to identify the organisms or their functions to perform effective environmental classification. Hence, we do not need large databases of annotated metagenomic reads (like the NCBI (nt) nucleotide database), which substantially decreases the amount of data we have to process. Furthermore, this makes it possible to exploit the organisms specific to each location, even if their genetic material is not included in the databases. Taking that into account, in our work, the microbiome fingerprint is defined as a set of DNA fragments (*k*-mers) derived from organisms living in a given city.

In the reported study, we exploit our CoMeta (Classification of Metagenomes) program [26], which allows for fast classification of metagenomic samples, and we apply it to classify the extracted unknown metagenomes to a set of collections of known samples. We employ an improved, yet unpublished version of CoMeta, which uses the *k*-mer databases built with the KMC 3 program [27]. We construct separate groups of metagenomic reads for each *city* to compare the samples on the basis of their similarity, measured directly in the space of the metagenomic reads. Moreover, we use the CoMeta program to cluster the samples based on their mutual similarities, which allows us to identify several groups that have been derived from the same origin. In addition to CoMeta, we have explored the possibility of using the Mash program [28] for determining the similarity between the samples—the classification scores obtained with CoMeta and Mash are reported and discussed in the paper.

## Materials and methods

### Metagenomic data

**Fig. 1:**
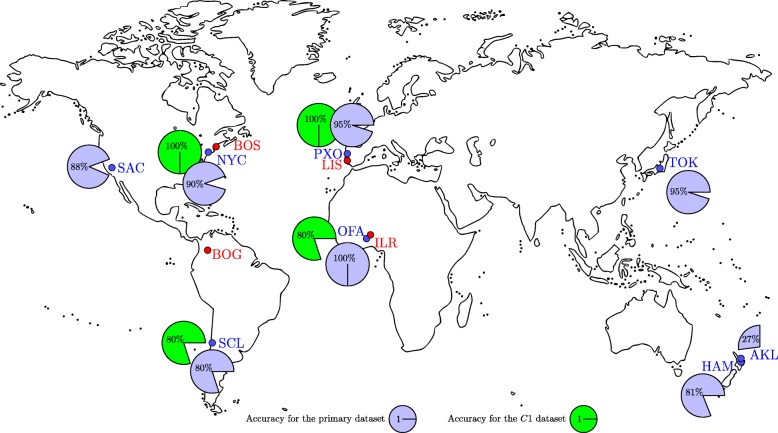
A map presenting the origin of the samples in the MetaSUB dataset. The eight cities marked with blue color are included in the primary dataset, and four cities marked with red color are the origins of the samples included in the *C*2 and *C*3 sets. On the map, we show the classification accuracies (obtained using the proposed method) for the cities from the primary dataset—blue indicates the scores for the primary dataset (based on leave-one-out cross validation), and green shows the scores for the *C*1 set (which includes samples from four cities out of eight from the primary dataset)

The MetaSUB Challenge embraces three complementary independent test sets and a *primary dataset* (i.e., the reference set with all the metadata provided, including geographical origin of the data). The characteristics of the samples in the primary dataset are provided in the Additional file 1. The samples in the first test set (*C*1) were acquired from a variety of surfaces in several different cities. For the CAMDA contest, the origin of the *C*1 samples was unknown, however it was stated that these locations are the same as for the samples from the primary dataset. The samples from the second set (*C*2) come from three cities that are not included in the primary dataset, and each city is represented by 12 samples (these groups were known for the contest, but the origin of each group remained unknown). Finally, the third set (*C*3) contains 16 samples, which were not grouped at all (also it was unknown from how many cities they were gathered). The geographic origin for all the samples in the test sets was published just before the CAMDA contest (the *C*3 samples originate from four cities, three of which are the same as those in *C*2). These three sets were compared with the annotated data from the primary dataset, available at the CAMDA 2018 website. The primary dataset contains 311 samples derived from eight cities in six countries (see Table 1 for details). A map presenting the cities of origin for all the samples is shown in Fig. 1.

**Table 1:** The content of the primary data set before and after removing human DNA fragments

ID	Country	City	#samples	Average #reads per sample	
				Original data	Without human DNA
SCL	Chile	Santiago	20	14,895,560	10,281,642
TOK	Japan	Tokyo	20	28,234,328	12,172,488
AKL	New Zealand	Auckland	15	4,929,497	4,849,711
HAM	New Zealand	Hamilton	16	6,073,774	5,999,711
OFA	Nigeria	Offa	20	35,469,676	34,936,176
PXO	Portugal	Porto	60	5,100,568	3,406,160
NYC	USA	New York	126	8,437,471	7,059,544
SAC	USA	Sacramento	34	25,153,713	22,627,578
Together			311	12,757,221	10,224,299

All files were delivered as compressed FASTQ files. After unpacking and converting to FASTA files (used in the analysis) the sizes were as follows: 492 GB for test sets and 1.44 TB for primary datasets. Information about the number of samples for each set with average number of reads is reported in Tables 1 and 2. In the tables, we report these numbers before and after removing the reads with human DNA fragments, which is discussed later in the paper.

**Table 2:** The test sets (*C*1, *C*2, and *C*3) before and after removing human DNA fragments

Metagenome sample →	*C*1 set	*C*2 set	*C*3 set
#samples	30	3×12	16
Average #reads per sample (original)	4,637,923	28,907,439	18,000,000
Average #reads (without human DNA)	3,871,596	25,082,590	15,027,017

### Data preprocessing

To prepare the data for classification, we construct *k*-mer databases and we preprocess the reads from each query sample.

Moreover, if the Mash program is used to estimate the similarity between the samples, we construct a *sketch* for each sample.

For each reference sample (to which the query samples are compared), we create a separate sample-level database, and then the databases created from samples that belong to the same class are combined together into one larger class-level database (so we end up with one database per class). We perform a similar operation for the Mash sketches—we combine the results for samples derived from the same class (i.e., a city). In the Mash program, the reads must be first sketched with *s* hashes (termed the *sketch size*). In order to easily combine the samples into one class, which is particularly useful for leave-one-out validation, we have created an auxiliary program for combining the files (obtained after sketching) into a single file—thus, multiple lists of hashes are joined into one list of hashes. Our program loads all the hashes (*s* hashes from each sample), then sorts them and saves a new set of *s* unique hashes for each class.

The *k*-mer databases (for the CoMeta program) are constructed using the KMC program, which extracts *k*-mers composed only of known nucleotides (‘A’, ‘C’, ‘T’, and ‘G’), and those that contain at least one ‘N’ symbol are ignored. The databases are subsequently filtered to reject the *k*-mers which appear less than *ci* times, as they may result from sequencing errors. We have considered two variants here: (*i*) filtering applied to the sample-level databases (before they are joined to form a class-level database) and (*ii*) filtering of the final class-level databases.

For the MetaSUB Challenge, most of the reads from the primary dataset and all reads from the test sets are paired-end. The CoMeta program determines the similarity between every read from a query sample and each database. Therefore, taking the above into account, we concatenate the paired-end sequences in order to obtain a single score for each read pair. The first read is rewritten, and a reverse complement of the second read is appended to it. These two fragments are separated from each other with a marker symbol (we use ‘N’ to differentiate it from the symbols that appear in the database of *k*-mers—note that KMC rejects all the *k*-mers that contain ‘N’). For example, if the first-end read is ACGT (usually much longer) and the second-end read is TTTC, then our output sequence is ACGTNGAAA. Afterwards, such a sequence is split into *k*-mers and compared with the database (the *k*-mers with ‘N’s extracted from the query read do not appear in the database, so they do not affect the computed similarity).

The majority of studies on metagenomes are focused on analysing the bacteria in an investigated sample [5, 9]. In some studies, also other kinds of microbiomes are included for analysis (like fungi, archaea, non-living viruses) [7]. Importantly, it can be expected that the MetaSUB samples acquired from different sites contain highly-similar fragments of the human genome. These human fragments rather do not help in the analysis, hence we decided to remove human DNA from the investigated samples. For this purpose, we used the kmc_tools software [29]. The file (GRCh38_latest_genomic.fna.gz) with the human reference genome was downloaded from the NCBI Website. For this file, we build a *k*-mer database using the KMC 3 program [27], and we subtract this database from every class-related database. In addition to that, we filter each query sample—if at least one human *k*-mer (*k*=24) appears in a read, then that read is removed from the sample. Information about the sizes of the samples before and after removing the human DNA fragments are reported in Tables 1 and 2. This operation allowed for reducing the sizes of the samples from the test sets by 1% to about 50%.

### Data classification

For classifying the metagenomic samples, we have adopted the methodology developed within our earlier study on forensic data analysis [30]. In the research reported here, we introduce several modifications, which include removing human fragments (as discussed earlier in the paper) and filtering infrequent *k*-mers from the databases (here, we consider two variants). We determine the similarity between metagenomic samples using our CoMeta program [26]. It has been designed for fast and accurate classification of reads obtained after sequencing entire environmental samples, and it allows for building a database without any restrictions. The similarity between the query read and each class (group) of the reference sequences is determined by counting the number of the nucleotides in those *k*-mers which occur both in the read and in the group (the algorithm is described in detail in [26]).

There are a number of other tools for comparing metagenomic data [6, 7, 31], which potentially may also be employed for classifying the metagenomic samples directly from the sequence similarity, without performing taxonomic or functional classification. In this paper (as mentioned earlier), we focus on checking whether such classification scheme is effective, rather than finding the best tool for comparing the metagenomic data. Nevertheless, in addition to employing CoMeta for this purpose, we decided to test the Mash program as an alternative tool which performs approximate matching.

**Fig. 2:**
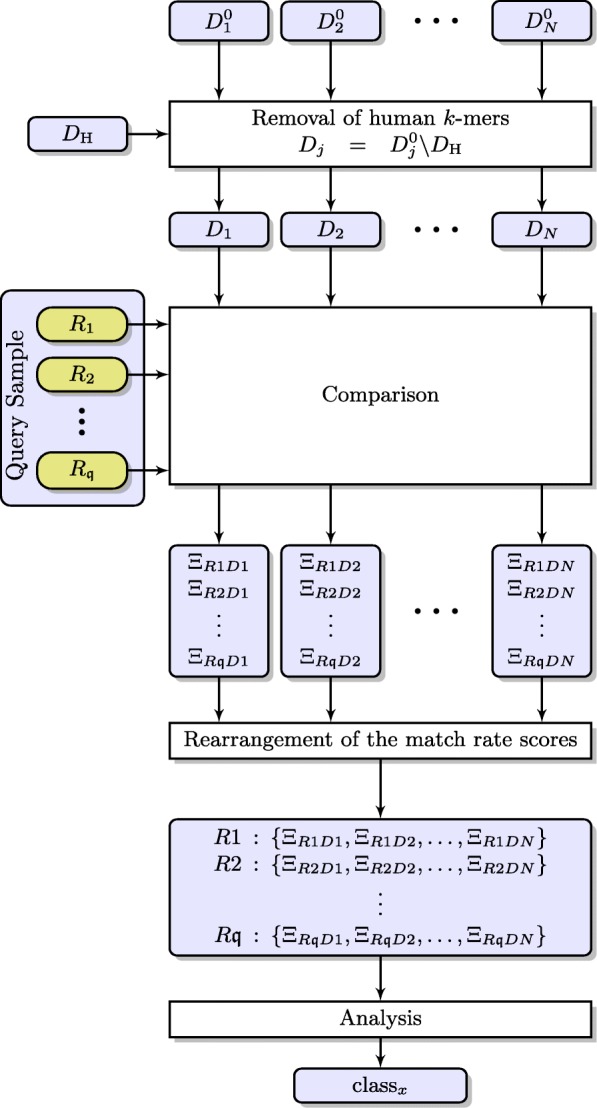
The processing pipeline for classifying metagenomic reads to one of the constructed classes. *D*_*H*_—*k*-mer database for the human reference sequence; $\left \{D^{0}_{1}, D^{0}_{2}, \dots, D^{0}_{N}\right \}$—*k*-mer databases from the original datasets for each of *N* classes; {*D*_1_,*D*_2_,…,*D*_*N*_}—*k*-mer databases after subtracted *D*_*H*_ for each of *N* classes; *R*_*i*_—an *i*th read from a query sample which includes $\mathfrak {q}$ reads; *Ξ*_*RiDj*_—a result of matching a *j*th read to the *i*th class (match rate score); *x*—one of the constructed classes; each blue block indicates data stored in a separate file

A simplified diagram of our classification scheme (using CoMeta) is shown in Fig. 2. At first, *N* groups (classes) containing reference sequences (reads) are created and the reads from the query sample are compared with them. For each *i*th class, the *k*-mer database ($D^{0}_{i}$) is built from the original datasets (before removing human fragments) using the KMC software. In addition, a *k*-mer database for the human reference sequences is built (termed *D*_H_). Subsequently, *D*_H_ is subtracted from each original *k*-mer database using the kmc_tools software ($D_{j}=D^{0}_{j} \backslash D_{\mathrm {H}}$). Each read *R*_*i*_ among $\mathfrak {q}$ reads derived from a query sample is compared against each class using CoMeta. We use only canonical *k*-mers (i.e., a lexicographically smaller item of the pair: the *k*-mer and its reverse complement), therefore there is no need to check the reverse complement of these reads. From the comparison, for each *i*th read and *j*th class, we obtain their mutual similarity value, termed the *match rate score* (*Ξ*_*ij*_). This value is a ratio of the number of the nucleotides in the *k*-mers which occur both in the read and in the database (associated with the class) to the length of the query read. A detailed algorithm for computing this value is reported in [26].

For each *i*th read, we create a list of match rate scores *R*_*i*_:{*Ξ*_*i*,1_,*Ξ*_*i*,2_,…,*Ξ*_*i*,*N*_}, and we analyze only these classes, whose *Ξ*’s are greater than or equal to a *similarity threshold*$\mathbb {T}$. We also take into account the number of classes which meet that condition—we ignore these reads, for which that number is larger than a *maximum class number threshold*$\mathbb {M}$. Formally, the *i*th read is skipped, if $\#\{D_{j}: \Xi _{ij} \geq \mathbb {T}\} > \mathbb {M}$, $1\leq \mathbb {M} \leq N$.

For example, let *N*=6, $\mathbb {M}=3$, $\mathbb {T}=50$, and the match rate score lists (for a sample composed of three reads) be *R*_1_: $\{30, \underline {80}, \underline {85}, \underline {50}, \underline {90}, 35\}$, *R*_2_: $\{20, \underline {90}, 0, 49, 0, 30\}$, and *R*_3_: $\{20, \underline {88}, \underline {90}, 0, 0, \underline {50}\}$ (the underlined values meet the condition $\Xi \ge \mathbb {T}$). Here, *R*_1_ does not meet the condition of the maximum number of classes (the number of underlined values is greater than $\mathbb {M}$), so *R*_1_ is ignored, and only *R*_2_ and *R*_3_ are further processed (*R*_2_ is matched with *D*_2_ and *R*_3_ is matched with *D*_2_, *D*_3_, and *D*_6_).

To determine the similarity of a sample (a set of reads) to each class, we process each read that meets the aforementioned conditions, and we cumulate the *similarity points* for each class. We consider three ways of computing these points:

- **simple sum:** each class gets 1 point for every matched read, no matter how many classes that read is matched to, and regardless of the differences between *Ξ*’s for individual classes. For our earlier example, *D*_2_ gets 2 pts, while *D*_3_ and *D*_6_ get 1 pt.
- **fractional sum:** each class gets (1/*n*) pt for an *i*th matched read, where $n=\#\{D_{j}: \Xi _{ij} \geq \mathbb {T}\}$ (*n*≥1 for matched reads). In our example, *D*_2_ gets 4/3 pt, while *D*_3_ and *D*_6_ get 1/3 pt.
- **weighted sum:** a *j*th class gets $\left (\Xi _{j} / \sum _{a \in A} \Xi _{a}\right)$ pt, where $A=\{j: \Xi _{ij} \geq \mathbb {T}\}$. In our example, *D*_2_ gets (1+88/(88+90+50))=1.39 pt, *D*_3_ gets (90/(88+90+50))=0.39 pt, and *D*_6_ gets (50/(88+90+50))=0.22 pt.

Finally, we normalize the value of collected similarity points by the number of reads in the query sample to obtain the similarities to all the classes, and the sample is classified to the class of the largest similarity. For our example, regardless of the way used for computing the similarity points, the query sample would be assigned to *D*_2_ (for the weighted sum approach, the similarities would be: 46.33% for *D*_2_, 13% for *D*_3_, 7.33% for *D*_6_, and 0% for *D*_1_, *D*_4_, and *D*_5_).

## Experimental validation

In this section, we present our experimental study performed using MetaSUB Challenge data to evaluate our method and compare it with other techniques. We outline the metrics used for evaluating the performance of investigated methods in “Evaluation methodology” section, the obtained results are briefly reported in “Results” section and discussed in detail in “Discussion” section.

### Evaluation methodology

To evaluate our method, we perform leave-one-out cross validation for the primary dataset. For the *C*1 test set, we classify the samples against the primary dataset to check whether they were assigned correctly. In both scenarios, for every *i*th class, we determine the number of correctly classified samples (*TP*_*i*_), predicted as belonging to that *i*th class, and the number of samples incorrectly labeled as belonging to that *i*th class (*FP*_*i*_). From these values, we compute *recall* (*true positive rate*):

$${TPR}_{i} = \frac{{TP}_{i}}{n_{i}}, $$

where *n*_*i*_ is the number of samples that belong to the *i*th class, *precision* (*positive predictive value*):

$${PPV}_{i} = \frac{{TP}_{i}}{{TP}_{i}+{FP}_{i}}, $$

and *overall classification accuracy*:

$${ACC} = \frac{\sum_{i}^{N} {TP}_{i}}{N_{s}}, $$

where $N_{s} = \sum _{i}^{N} n_{i}$ is the total number of samples.

### Results

Our experimental study has been divided into three main parts: *(i)* determining the values of the hyper-parameters of our method, *(ii)* comparison of our method against other techniques reported in the literature, and *(iii)* classification of samples, whose origin was not covered by the primary dataset. For the first two parts, we exploited the primary dataset and the *C*1 test set, while for the third part, the *C*2 and *C*3 test sets were used. The performance of the Mash program that we considered as an alternative to CoMeta, was verified using the primary set and the *C*1 test set.

In Tables 3 and 4, we show how our method performs for the primary dataset (based on leave-one-out cross validation, performed for 311 samples) depending on whether the infrequent *k*-mers are filtered at the class-level and sample level, respectively. For each case, we investigated three different techniques for computing the similarity scores, namely simple sum, fractional sum, and weighted sum. We report the overall classification accuracy for different values of thresholds $\mathbb {T}$ and $\mathbb {M}$. In order to verify that the coefficients $\mathbb {T}$ and $\mathbb {M}$ have similar impact on the *C*1 test set, the same experiment was performed for that test set (see Tables 5 and 6). For *C*1, the samples were classified using the databases constructed from the primary dataset, hence cross validation was not performed (it is worth noting that *C*1 is much smaller, as it contains only 30 samples). Based on Table 4, the remaining experiments reported in the paper were performed for sample-level filtering (if not stated otherwise), using weighted sum, and with $\mathbb {T}=50\%$ and $\mathbb {M}=8$.

**Table 3:**
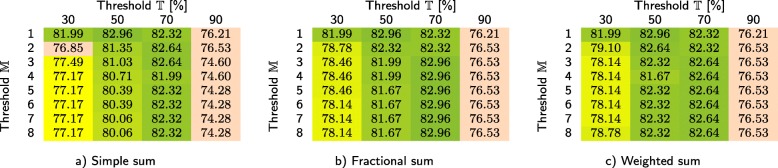
Classification accuracy obtained for the primary dataset using our method with class-level filtering at *ci*=4

We report the scores for three approaches to cumulating the similarity points for a sample: a) simple sum, b) fractional sum, and c) weighted sum, each for different values of threshold $\mathbb {T}$ and maximum number of classes that a single read can be classified to ($\mathbb {M}$)

**Table 4:**
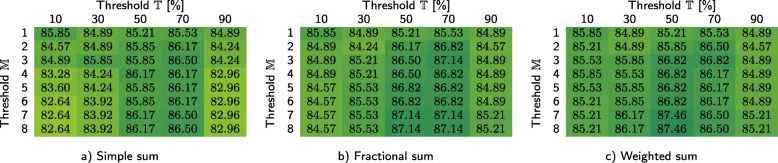
Classification accuracy obtained for the primary dataset using our method with sample-level filtering at *ci*=4

We report the scores for three approaches to cumulating the similarity points for a sample: a) simple sum, b) fractional sum, and c) weighted sum, each for different values of threshold $\mathbb {T}$ and maximum number of classes that a single read can be classified to ($\mathbb {M}$)

**Table 5:**
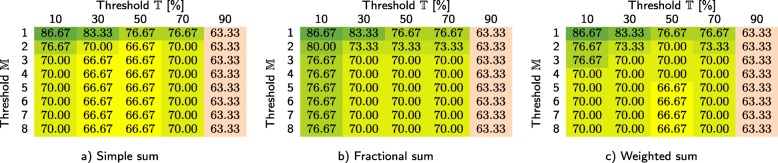
Classification accuracy obtained for the *C*1 test set using our method with class-level filtering at *ci*=4

We report the scores for three approaches to cumulating the similarity points for a sample: a) simple sum, b) fractional sum, and c) weighted sum, each for different values of threshold $\mathbb {T}$ and maximum number of classes that a single read can be classified to ($\mathbb {M}$)

**Table 6:**
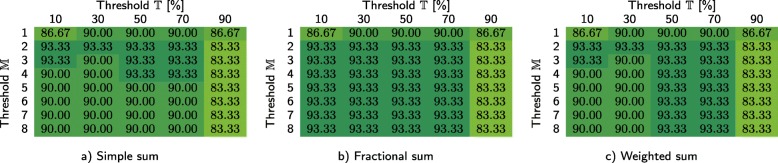
Classification accuracy obtained for the *C*1 test set using our method with sample-level filtering at *ci*=4

We report the scores for three approaches to cumulating the similarity points for a sample: a) simple sum, b) fractional sum, and c) weighted sum, each for different values of threshold $\mathbb {T}$ and maximum number of classes that a single read can be classified to ($\mathbb {M}$)

Confusion matrix for the primary dataset obtained based on leave-one-out cross validation is presented in Table 7. For each row, we show samples from a single location, classified to eight classes, created from the remaining 310 samples (the correct results are positioned on the diagonal). Performance metrics obtained from this matrix are reported in Table 8 (three bottom rows). We also show the scores obtained with class-level filtering, and for the Mash program, we consider three values of the sketch size (as in CoMeta, the *k*-mer length is 24). In both cases, we use leave-one-out cross validation. Moreover, we quote the results published in other papers. The evaluation methodology varied across these works. Most of them performed cross validation for the primary dataset [22–24] (including 10-fold [32] and leave-one-out [25] approaches). However, in some studies, only a subset of the primary dataset was analyzed, so we provide the number of samples (*N*_*s*_) in the table. All the papers report classification accuracy and most of them provide precision (*PPV*) and recall (*TPV*) for the individual classes. It is worth noting that our validation methodology for the primary dataset is identical to that adopted by Zhu [25], and no additional data were published after the CAMDA 2018 competition concerning the primary dataset.

**Table 7:** Confusion matrix for the primary dataset obtained using our method with sample-level filtering, similarity points computed using weighted sum, with $\mathbb {T}=50\%$ and $\mathbb {M}=8$

Predicted →									
Tested *↓*	AKL	HAM	NYC	OFA	PXO	SAC	SCL	TOK	ALL
AKL	**4**	6	4	0	1	0	0	0	15
HAM	2	**13**	1	0	0	0	0	0	16
NYC	1	1	**113**	11	0	0	0	0	126
OFA	0	0	0	**20**	0	0	0	0	20
PXO	0	0	1	0	**57**	0	0	2	60
SAC	0	0	3	0	0	**30**	1	0	34
SCL	0	0	1	0	3	0	**16**	0	20
TOK	0	0	0	0	1	0	0	**19**	20
ALL	7	20	123	31	62	30	17	21	311

The diagonal values in bold indicate the correct results

**Table 8:** Scores obtained for the primary dataset using cross validation

		AKL	HAM	NYC	OFA	PXO	SAC	SCL	TOK	Total
Ryan [21]	#correct	7	10	25	20	60	16	18	20	$\sum =176$
	*PPV*	0.54	0.56	0.96	0.95	0.98	1	1	1	*N*_*s*_=193
	*TPR*	0.47	0.63	0.96	1	1	1	0.9	1	*ACC*=0.912
Sanchez et al. [24]	#correct	9	11	110	17	60	34	17	20	${\sum }=278$
	*PPV*	0.69	0.73	0.95	0.89	1	0.83	0.89	0.71	*N*_*s*_=311
	*TPR*	0.6	0.69	0.87	0.85	1	1	0.85	1	*ACC*=0.894
Harris et al. [32]	—	—	—	—	—	—	—	—	—	*N*_*s*_=*N*/*A*
										*ACC*=0.897
Walker and Datta [22]	*TPR* (median)	0.6	0.62	0.58	0.95	0.87	0.76	0.3	0.7	*N*_*s*_=211
	—	—	—	—	—	—	—	—	—	*ACC*=0.71
Zhu [25]	#correct	5	3	114	14	51	31	17	15	${\sum }=250$
	*TPR*	0.33	0.19	0.9	0.74	0.85	0.91	0.85	0.75	*N*_*s*_=310
										*ACC*=0.81
Chierici et al. [23]	—	—	—	—	—	—	—	—	—	*N*_*s*_=311
										*ACC*=0.894
Our method using Mash	#correct	15	15	50	20	60	31	19	20	${\sum }=230$
*sketch* *size*=1000	*PPV*	0.34	0.26	1.00	0.67	1.00	1.00	1.00	1.00	*N*_*s*_=311
	*TPR*	1.00	0.94	0.40	1.00	1.00	0.91	0.95	1.00	*ACC*=0.740
Our method using Mash	#correct	15	16	42	20	60	34	20	20	${\sum }=227$
*sketch* *size*=10000	*PPV*	0.65	0.18	1.00	0.83	1.00	1.00	1.00	1.00	*N*_*s*_=311
	*TPR*	1.00	1.00	0.33	1.00	1.00	1.00	1.00	1.00	*ACC*=0.730
Our method using Mash	#correct	15	16	44	20	60	34	19	20	${\sum }=228$
*sketch* *size*=100000	*PPV*	0.60	0.18	1.00	1.00	1.00	1.00	1.00	1.00	*N*_*s*_=311
	*TPR*	1.00	1.00	0.35	1.00	1.00	1.00	0.95	1.00	*ACC*=0.733
Our method using CoMeta	#correct	4	12	116	20	37	34	13	20	${\sum }=256$
(class-level filtering)	*PPV*	0.67	0.63	0.92	0.74	1.00	0.97	1	0.42	*N*_*s*_=311
	*TPR*	0.27	0.75	0.92	1.00	0.62	1.00	0.65	1.00	*ACC*=0.823
Our method using CoMeta	#correct	4	13	113	20	57	30	16	19	${\sum }=272$
(sample-level filtering)	*PPV*	0.57	0.65	0.92	0.65	0.92	1.00	0.94	0.9	*N*_*s*_=311
	*TPR*	0.27	0.81	0.9	1.00	0.95	0.88	0.8	0.95	*ACC*=0.875

We report the number of correctly classified samples (#correct), precision (*PPV*), and recall (*TPR*) for each class, as well as the overall accuracy (*ACC*). Some of the values are missing, as they were not reported in the referenced papers. Also, we show the number of samples (*N*_*s*_), as in some works, the results for a subset of all of *N*_*s*_=311 samples were reported

In Table 9, we report the similarities (defined earlier in “Data classification” section) between every sample in the *C*1 test set and each class from the primary dataset, obtained using our method with the CoMeta program. Each sample is classified to the class with the highest similarity. Final classification outcomes obtained with different methods for the *C*1 test set are presented in Table 10, and they are summarized in Table 11. As for the primary dataset, we quote the scores that were reported in the papers focused on the MetaSUB Challenge.

**Table 9:**
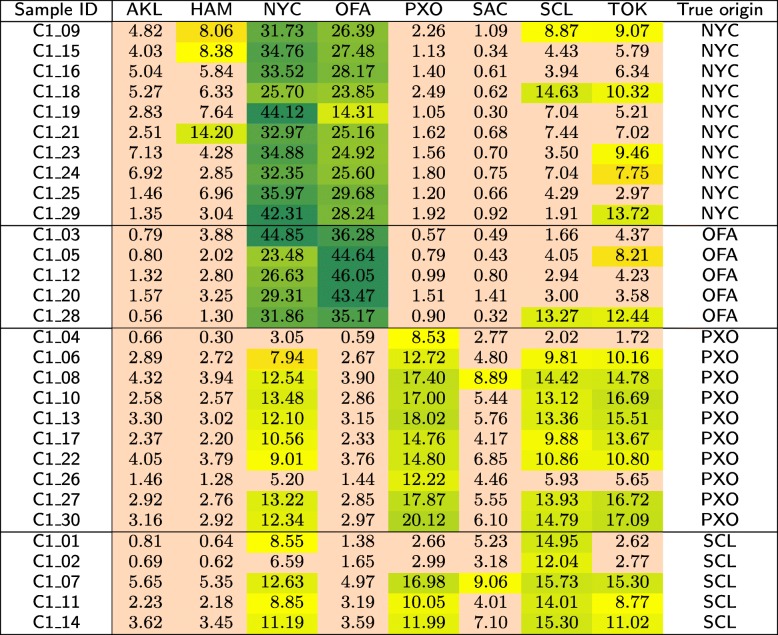
Similarities (in %) of the samples in the *C*1 test set to the individual classes from the primary dataset, obtained using our method

**Table 10:**
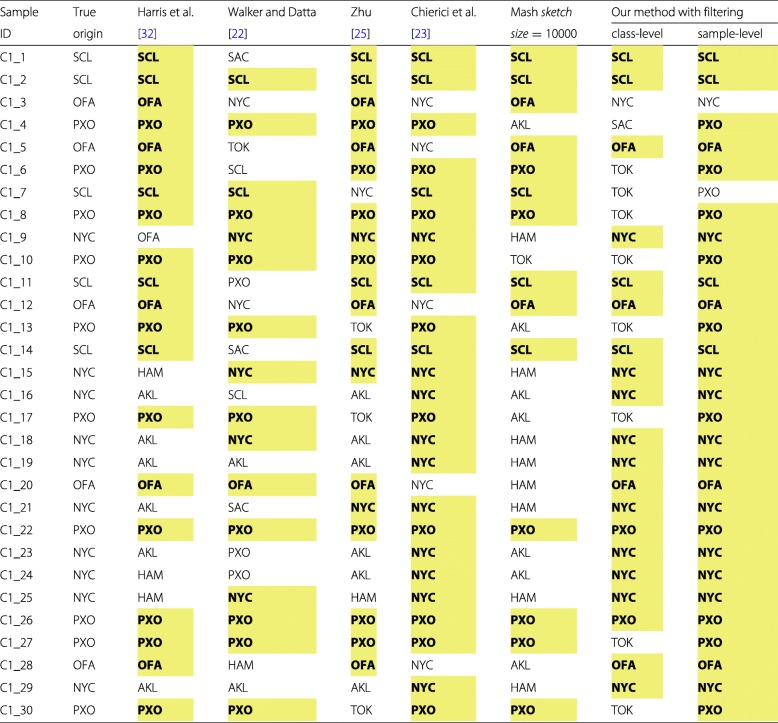
Detailed classification outcomes obtained using different methods for the *C*1 test set. The correct results are highlighted

**Table 11:** Classification scores obtained for the *C*1 test set using different methods

		NYC	OFA	PXO	SCL	Overall accuracy
Harris et al. [32]	#correct	0	5	10	5	
	*PPV*	—	0.83	1.00	1.00	*ACC*=0.667
	*TPR*	0.00	1.00	1.00	1.00	
Walker and Datta [22]	#correct	4	1	9	2	
	*PPV*	0.67	1.00	0.75	0.50	*ACC*=0.533
	*TPR*	0.40	0.20	0.90	0.40	
Zhu [25]	#correct	3	5	7	4	
	*PPV*	—	5.00	0.58	1.00	*ACC*=0.633
	*TPR*	0.30	1.00	0.70	0.80	
Chierici et al. [23]	#correct	10	0	10	5	
	*PPV*	0.67	—	1.00	1.00	*ACC*=0.833
	*TPR*	1.00	0.00	1.00	1.00	
Our method using Mash	#correct	0	3	4	2	
*sketch* *size*=1000	*PPV*	—	1	1	0.5	*ACC*=0.300
	*TPR*	0	0.6	0.4	0.4	
Our method using Mash	#correct	0	3	6	5	
*sketch* *size*=10000	*PPV*	—	1	1	1	*ACC*=0.467
	*TPR*	0	0.6	0.6	1	
Our method using Mash	#correct	0	3	5	4	
*sketch* *size*=100000	*PPV*	—	1	1	1	*ACC*=0.400
	*TPR*	0	0.6	0.5	0.8	
Our method using CoMeta	#correct	10	4	2	4	
(class-level filtering)	*PPV*	0.91	1.00	0.91	1.00	*ACC*=0.667
	*TPR*	1.00	0.80	1.00	0.80	
Our method using CoMeta	#correct	10	4	10	4	
(sample-level filtering)	*PPV*	0.91	1.00	1.00	1.00	*ACC*=0.933
	*TPR*	1.00	0.80	0.20	0.80	

We report the number of correctly classified samples (#correct), precision (*PPV*), and recall (*TPR*) for each class, as well as the overall accuracy (*ACC*)

The *C*2 test set is composed of three groups (*C*2_*C*1, *C*2_*C*2, and *C*2_*C*3), each of which contains 12 samples acquired from the same geographical location. These locations were made publicly known after closing the MetaSUB Challenge—these were Ilorin (Nigeria), Lisbon (Portugal), and Boston (USA). In Tables 12, 13, and 14, we show the similarities between the samples in *C*2 and the classes from the primary dataset.

**Table 12:**
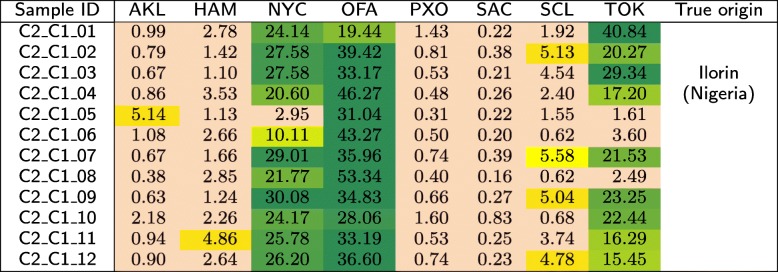
Similarities (in %) of the samples that originate from Ilorin (Nigeria) in the *C*2 test set to the individual classes from the primary dataset, obtained using our method

**Table 13:**
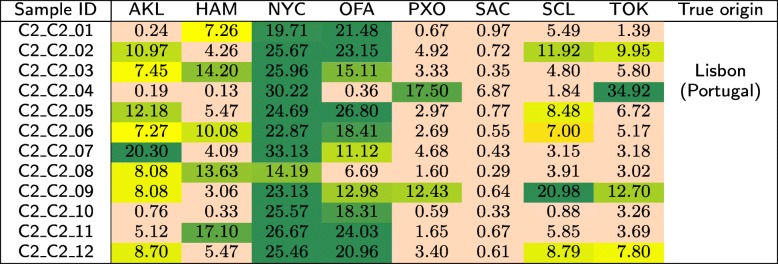
Similarities (in %) of the samples that originate from Lisbon (Portugal) in the *C*2 test set to the individual classes from the primary dataset, obtained using our method

**Table 14:**
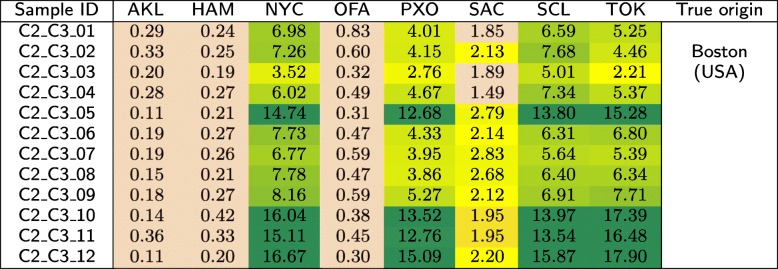
Similarities (in %) of the samples that originate from Boston (USA) in the *C*2 test set to the individual classes from the primary dataset, obtained using our method

In Table 15, we show the mutual similarities between 16 samples in the *C*3 test set, which were derived from four cities (they include three cities covered by *C*2 and Bogota in Colombia). For the MetaSUB Challenge, the number of locations and their relation with other sets were unknown, so this task consisted in clustering of the samples. Subsequently, we normalized the similarities for each sample (i.e., each row in Table 15), so that the maximum similarity for each sample equals 100%, and we reordered the samples to identify the clusters (Table 16). After clustering, we measured the similarity between the samples in *C*3 with the classes from the primary dataset and from the *C*2 set. The obtained similarity scores are reported in Table 17.

**Table 15:**
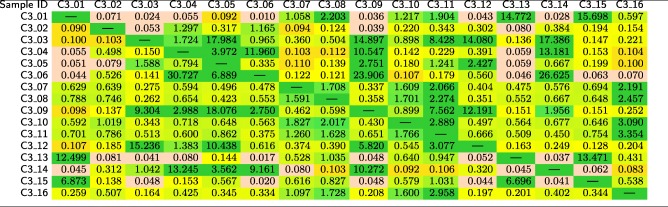
Mutual similarities (in %) between the samples in the *C*3 test set, obtained using our method

**Table 16:**
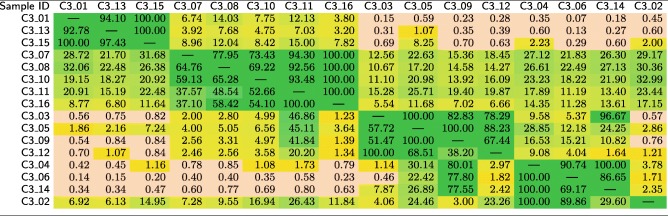
Mutual similarities (in %) between the samples in the *C*3 test set, obtained using our method, normalized independently for each row

The samples were sorted manually to identify four clusters (cluster 1: *C*3_01, *C*3_13, and *C*3_15, cluster 2: *C*3_07, *C*3_08, *C*3_10, *C*3_11, and *C*3_16, cluster 3: *C*3_03, *C*3_05, *C*3_09, and *C*3_12, and cluster 4: *C*3_04, *C*3_06, *C*3_14, and *C*3_02)

**Table 17:**
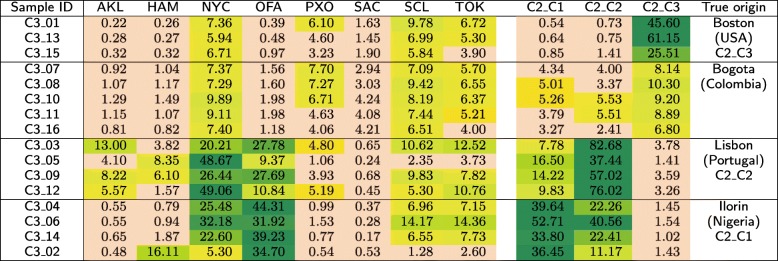
Similarities (in %) of the samples that originate in the *C*3 test set to the individual classes from the primary dataset and from the *C*2 test set, obtained using our method

Three out of four ground-truth origins were identical to these of the samples from the *C*2 set

The time needed to build a *k*-mer database composed of 10^6^ reads was ca. 11.5 s (0.05 s to read 10^6^ 24-mers). To compare a sample against a *k*-mer database using CoMeta (to obtain match rate scores for every read), around 47 s were required for every 10^6^ reads (an average time for the databases in the MetaSUB Challenge data). The time needed to obtain the final similarity of a sample (for 10^6^ reads) to all of the eight classes, was ca. 3.75 s.

### Discussion

Our experiments on the primary dataset allowed us to determine the best settings and values of the hyper-parameters, as well as to analyze the sensitivity of our method. From Tables 3 and 4, it can be seen that the sample-level filtering of infrequent *k*-mers is definitely better than if the databases are filtered at the class level. Probably this is due to the fact that during the sample-level filtering, only these *k*-mers are selected, which occur at least a few times in a single sample (which reduces the risk of selecting *k*-mers present due to sequencing errors). During the class-level filtering, single erroneous *k*-mers can be accumulated, as the databases at the class level are much larger. Possibly, the *ci* parameter should depend on the database size, but this would have to be verified.

The differences between three approaches towards computing the similarity points allow us to conclude that it is beneficial to take into account the number of classes that each read is classified to (fractional sum and weighted sum are better than simple sum). The sensitivity of our method to the thresholds $\mathbb {T}$ and $\mathbb {M}$ is rather low—in general, the results are best, if $\mathbb {T}$ is around 50% and $\mathbb {M}=8$ (i.e., the number of classes in the primary dataset). Importantly, the observations made for the primary dataset were also confirmed by the results obtained for the *C*1 test set—even though it is much smaller, the same tendencies emerge here (Tables 5 and 6).

From Table 8, it can be seen that our method (with CoMeta employed) is competitive with other techniques with overall accuracy at 0.875, compared with the best result of 0.912 [21] and the lowest of 0.71 [22]. However, the best score was reported for an unspecified subset of the primary dataset (with *N*_*s*_=193 samples). The best scores for the entire primary dataset (*N*_*s*_=311) were reported in [23, 24] with an accuracy of 0.894. It is worth noting that the scores quoted for other methods were reported at the CAMDA 2018 conference, and it may be expected that these initial results will be further improved. On the other hand, the primary set was fully described before CAMDA 2018, so we do not benefit from any additional information. When we use Mash instead of CoMeta for comparing the samples, the results of classification are significantly worse. Mash determines the similarity between the samples by counting the number of *k*-mers found in both samples. CoMeta counts the sum of matched fragments, (composed of *k*-mers), which makes it more resistant to accidental similarities of short fragments. An important advantage of our approach is that contrary to these alternative methods, we do not perform taxonomic or functional classification. Thus, for comparing the samples we can exploit even those fragments of sequences which are not covered by the reference databases.

The results obtained using our method for the *C*1 data set are much better than those reported in other works (Table 11), but it must be taken into account that the ground-truth data were published only after closing the MetaSUB Challenge, which puts us in a privileged position here. Actually, in our submission to CAMDA 2018 [33], we identified correctly 19 out of 30 samples (hence the accuracy was of 0.633), but no infrequent *k*-mer filtering was performed there, and also we did not remove human DNA from the samples. In the approach proposed here, we misclassify only 2 out of 30 samples (see Table 10), but we strongly benefit from information that each sample in *C*1 belongs to one of the known classes (that was clear before CAMDA 2018). It can be seen from Table 9 that the values of highest similarities in each row differ much among themselves. Thus, it would be difficult to establish a cut-off threshold required for open-world classification (when it is unknown whether a sample originates from the places covered by the reference set). Our approach with Mash used instead of CoMeta returned the worst results. While the scores for the primary dataset obtained using Mash are similar to the classification results returned by other methods, for the *C*1 test set they are extremely poor.

For the *C*2 test set, it can be seen from Table 12 that the first group of samples was most similar to Offa (Nigeria), so in our submission to CAMDA 2018 [33], we suspected that the samples originated from Africa. This was correct, as the samples were derived from Ilorin in Nigeria. Surprisingly, the samples that originate from Lisbon (Portugal) are most similar to New York (USA) and Offa (Nigeria), while being little similar to Porto (Portugal), which is geographically the closest to Lisbon (see Table 13). The samples acquired in Boston (USA) were similar to several classes, including New York, Porto, Santiago de Chile, and Tokyo (see Table 14). Apparently, the geographical neighborhood is not the most important factor influencing the similarity between metagenomic samples. It may be noticed that apart from the Nigerian cities, these are large metropolises with many people travelling around, which may affect the metagenomic fingerprint of these locations. Therefore, it may be an interesting research direction to analyze the differences between these databases to identify a set of unique *k*-mers that would work as a signature of a certain location.

From Table 16, it can be observed that the samples in the *C*3 test set form four clusters (we identified the same clusters in our CAMDA submission [33]), and they reflect the ground-truth origin of the samples, as shown in Table 17. For clustering, the sample-wise normalization helped much (compare Table 15 with Table 16), and it was not difficult to identify the clusters manually. Nevertheless, for more samples, it would be necessary to automate the clustering process, for example relying on bicluster induction that can be performed for discrete [34] and continuous data [35]. It can also be seen from Table 17 that the *C*3 samples are correctly classified to the databases constructed from the *C*2 set, which once again confirms that our method can effectively be used for classifying metagenomic data.

## Conclusions

In this paper, we introduced a new method for environmental classification of metagenomic reads to the reference groups. A significant advantage of our approach lies in determining the similarity between the samples at the read level, without the necessity to understand the contents of these samples. The results of our experimental study indicate that our approach is competitive with other methods that are based on taxonomic or functional classification of each sample, which makes them dependent on large databases of annotated reads. We investigated two different programs (CoMeta and Mash) for comparing the samples, and we found CoMeta much more appropriate for dealing with the investigated cases. Overall, we have demonstrated that environmental classification of metagenomic data is feasible without using such large datasets.

The reported experimental results indicated several limitations of the proposed method that can be addressed in our future research. First of all, the maximum values of the similarity scores vary much across the classes, so they would not be suitable for open-world classification. Possibly, some normalization could be helpful here as a postprocessing step. Furthermore, the results for the *C*2 set showed that geographical neighborhood does not necessarily imply similarity between the metagenomic samples—in the test sets, there were three pairs of cities located near each other: Boston with New York, Lisbon with Porto, and Offa with Iloris. Only for the last pair, we observed high similarity between the samples. This would have to be investigated whether the similarity measured at the taxonomic or functional level between these samples allows for obtaining better results in this case. A potentially interesting direction of future research would be to investigate the influence of the dataset characteristics specific for particular locations (such as microbial diversity or read depth) on the classification accuracy. Also, it could be explored more deeply how the preprocessing affects the classification accuracy—this may include checking the influence of removing human DNA or filtering infrequent *k*-mers using different values of *ci*, as well as tuning the value of *k* (i.e., the length of *k*-mers). Finally, for performing clustering of metagenomic samples, it may be interesting to exploit biclustering so as to make the reported approach scalable.

Our ongoing work is focused on enhancing our classification rules to consider both the similarities, as well as the dissimilarities between the samples. We plan to construct differential databases, which could be used as specific markers of particular locations. We intend to investigate whether this would help in understanding the surprising classification results observed for the *C*2 test set. Furthermore, we will employ the developed method for other datasets to check whether it is suitable for solving different kinds of metagenomic classification problems.

## Reviewers’ comments

### Reviewer 1, Eran Elhaik, Ph.D.

*In this study, the authors propose a new method to identify the geographical and surface of origins of microbiome samples. This method is applied to the MetaSUB database, as part of the MetaSUB Forensics Challenge of the CAMDA 2018 conference. It is very difficult to evaluate the method proposed by the authors since the manuscript is so poorly written. I hope that the authors would use my comments to improve their work.*

#### Detailed comments and responses (major recommendations)

1. *The abstract and the whole paper should be written succinctly. There is much repetition, use of long sentences, and marketing type of comments that are unwarranted (“Therefore, this analysis can help answer a variety of questions about the place from where the samples have been derived”). I expect a reduction of at least 25% in the size of both.*
  **Response:** Thank you for pointing that out—the abstract, as well as some parts of the paper were too long indeed. Also, we have carefully reviewed the paper to remove the statements which are unwarranted.
2. Figure 1*is unclear. There are no “red boxes” line 44, page 4.*
  **Response:** Thank you very much for drawing our attention to that. We have changed that figure alongside its caption and description in the text.
3. *Add more figures. For example, a figure with a map showing the origin of the samples with pie chart in each location showing what % were successfully predicted to those locations.*
  **Response:** We have modified Fig. 1 (which became Fig. 2 in the revised manuscript) and added the requested map (Fig. 1 in the revised manuscript). Also, we improved the way of presenting the data in the tables.
4. *The entire paper is completely deviant of any results or statistical analyses. This is not how research papers are written.*
  **Response:** For the revised manuscript, we substantially extended our experimental study and we analyse our method quantitatively.
5. *Did you consider using other methods? Maybe they work better? The paper would be far more convincing if you’d compare your method to other methods. I realize this involves more work, but this would markedly improve the paper. As it is, we have an unverified method, with some unclear performances, and we don’t know how other methods perform.*
  **Response:** Thank you for this comment. As many authors who contributed to the MetaSUB Challenge reported classification scores for the reference database containing 311 samples (using cross validation), we have also performed an experiment following the same protocol. This has allowed us to compare with other methods quantitatively, and these scores are reported in the paper.
6. *The authors adopted a strategy where the authors should dig the results from their numerous tables. That’s a poor habit. Put the results clearly in the manuscript.*
  **Response:** Thank you, the results were indeed not easy to analyze. We have reviewed the way we present our experimental results and we hope that this is much clearer now.

#### Detailed comments and responses (minor issues)

1. *“Importantly, the existing methods for environmental classification are based on taxonomic or functional classification which require large databases of annotated gene sequences” – when you say “the existing methods” can you be more specific? Also, can you focus on biogeography as this is the main focus of the paper.*
  **Response:** In the revised manuscript, we briefly review other papers which perform environmental classification, and we compare our results with the works submitted to CAMDA 2018.
2. *“The reported research was focused on verifying the reliability” – not verifying, testing. You don’t know that it works.*
  **Response:** We agree with that comment.
3. *“In our work, we showed that our approach” why do you need so many “our XX”? just say that “We showed that our approach...” there are many instances like that.*
  **Response:** Thank you for drawing our attention to that.
4. *“troublesome” from the abstract. Can you be more specific? Provide numbers!*
  **Response:** We have shortened the abstract and this phrase is no longer in the paper. Also, we have reviewed the manuscript to remove any ambiguities.
5. *The manuscript is full of typos. Commas are missing. Authors should use past tense when appropriate.*
  **Response:** We have carefully reviewed the manuscript and we hope that the language has been improved.
6. *there are many works which → many studies*
  **Response:** Thank you, we have changed that.
7. *remove “The paper is organized as follows” its obvious*
  **Response:** We agree that this paragraph was redundant, hence it was removed.
8. *I don’t understand the method. Why 2N’s are added in the middle and not 3 or 4?*
  **Response:** We have clarified that description—generally, it is important to separate a forward fragment from the backward one, and at least a single symbol can be inserted (so that the *k*-mers spanning over these two fragments are not matched with anything in the database). Actually, a single ’N’ would be sufficient, and we changed the description accordingly.
9. *First sentence of the methodology: This brief explanation about other methods should be expanded and go into the introduction? The justification for using the authors’ method should also be expanded.*
  **Response:** Thank you, we have restructured that description (we have also renamed it to “Data classification” section). The other methods are now described in “Background” section.
10. *What are the colors in Table* 7? *Table* 18 *and other tables. What is the true location? Why are there 1, 2, or 3 results? What is the threshold?*

**Table 18:** The number of unique *k*-mers in the class-level databases extracted from the primary dataset (for *k*=24) after filtering infrequent *k*-mers (with *ci*=4) from (i) sample-level databases and (ii) class-level databases

Class name	Class-level filtering	Sample-level filtering
Chile, Santiago	3,330,241,847	1,947,678,404
Japan, Tokyo	6,179,603,359	3,436,570,406
New Zealand, Auckland	586,168,771	567,504,772
New Zealand, Hamilton	897,549,433	845,417,208
Nigeria, Offa	3,293,428,857	2,833,690,965
Portugal, Porto	3,793,750,265	3,108,855,323
USA, New York	7,413,034,106	4,252,342,215
USA, Sacramento	2,413,540,643	599,036,464
  **Response:** In the revised manuscript, we have created most of the tables from scratch.

### Reviewer 2, Alexandra Bettina Graf

#### Initial submission

#### Reviewer summary

1. *The authors apply a k-mer approach using a previously published program (CoMeta) and methodology, to predict the location of metagenome samples of unknown origin in the frame of the CAMDA challenge. The samples consisted of the following datasets:*
  *Primary Dataset: 311 known samples from 8 cities in 6 countries*
  *C1 Sample Set: different cities and surfaces; unknown but selected from cities from the trainings set (primary dataset).*
  *C2 Sample Set: Samples from 3 cities that are not included in the training set (primary dataset). 12 samples/city.*
  *C3 Sample Set: 16 samples of unknown origin.*
  *Although the method is intriguing, at the moment the paper lacks objective measurements to evaluate the presented method against other available prediction methods.*
  **Response:** Thank you very much for this comment. In the revised manuscript, we compare our method with several other techniques that were used to approach the MetaSUB Forensic Challenge. As most of these studies report the scores for the tests on the primary dataset based on cross validation, we have performed the tests following the leave-one-out approach.
2. *I would like to see a more detailed analysis of the robustness and accuracy of the method. The authors work with datasets of which the ground truth is known so they can calculate the accuracy of their method.*
  **Response:** Thank you. We provide quantitative scores to summarize the qualitative results presented in our original manuscript. Also, we investigate the influence of several hyper-parameters on the classification accuracy.
3. *Did the authors test how their method is influenced by different parameters of the datasets, like microbial diversity or read depth (this will also be influenced by the human read content). Are there parameters of metagenome datasets that influence the accuracy of the method? Is there maybe a pattern in the data from cities which could correctly be predicted and data from cities that were not correctly predicted.*
  **Response:** Thank you for raising this idea. We have tried to analyse the *k*-mer histograms to determine the read depth, but we have not managed to reach any meaningful conclusions. As this may be an interesting direction for future research, we mention that in the final section.

#### Detailed comments and responses (major recommendations)

1. *The introduction reads unspecific and disconnected, and it is not clear what the authors want to describe.*
  **Response:** We have revised the manuscript carefully and restructured the unclear parts of the text.
2. *In reference 3,4 are tools to bin assembled contigs, assembly per-se does not use reference genomes, but for taxonomic or functional analysis one still needs to compare with known data or models.*
  **Response:** We have clarified that description and in the revised manuscript, we note that binning is used as a preprocessing step that precedes classification.
3. *There is nothing similar between reference 5, which is based on long reads, although they do use mock communities to evaluate their method, and Gerner et al. which developed a method for in-silico artificial communities against which to validate metagenome approaches.*
  **Response:** Thank you for drawing our attention to that—we have changed the description to be more specific.
4. *Zolfo et al., analyses the same CAMDA dataset as Gerner et al., but apart from that there is no similarity in method to Gerner et al. or Hudson et al.*
  **Response:** We have changed that description in the revised version.
5. *Removal of human DNA is a standard procedure in the analysis of metagenomes. The analysis will be strongly influenced by the amount of human DNA in the sample. As also seen by the authors, the amount of human DNA can be significant. It is often seen that a sample includes human variants, which are not in the reference genome, hence they would not be removed in the process. Could the presence the remaining human DNA cause a negative effect on the analysis?*
  **Response:** Human DNA may introduce some noise to the data, while increasing the size of the datasets and affecting time performance. It was confusing in the original submission, as we presented incomplete results without removing human DNA (which we presented at CAMDA 2018). In the revised paper, we report only the results obtained after removing human DNA.
6. *Did the authors see a correlation between content of human DNA and prediction accuracy? I would implore the authors to provide more information about the parameters of the dataset, and the behaviour of their method. Especially in view of a significant amount of wrong/unprecise predictions. For the C1 dataset, 23% of their predictions were incorrect, if one includes unspecific predictions (where more then one city was predicted) the value rises to 40%. For the C2 dataset only one of the three sets was predicted to be at least in the same country. For the C3 dataset it looks like the method is consistent in the prediction (when compared to the results for C2), but assigns incorrect locations.*
  *Were all datasets metagenome datasets, or also Amplicon?*
  *Did they have the same read length? Similar quality? Similar read depth?*
  *Were the reads trimmed or otherwise pre-processed, if so how?*
  *All of these factors can influence the k-mer content.*
  **Response:** We agree that it would be very interesting to explore how the properties of the metagenomic datasets affect the prediction accuracy (including removal of human DNA from the samples), and this is an interesting direction for future work. Answering the specific questions, there was no information provided on whether the sequences were amplified. The read lengths are generally uniform in majority of the samples (we report these lengths in a table attached as an Additional file 1), but there were also samples with varied read length. We have described how we preprocess the reads (actually, we do not trim them).
7. *The paper would also greatly benefit from the inclusion of other datasets and the comparison with other prediction approaches, in order to get a better picture of the performance of their method.*
  *How does the method perform with other datasets (e.g. Kawulok & Kawulok, 2018)?*
  *Or even more importantly how does it compare to other prediction methods in terms of prediction accuracy?*
  **Response:** In the revised manuscript, we still focus on the MetaSUB data, however, we have extended the analysis and added comparisons with other methods. As most of submissions to CAMDA 2018 report the scores for the primary dataset adopting leave-one-out cross validation, we have also performed that experiment. This allowed us to investigate the sensitivity of our method to its hyper-parameters and to compare its performance with other CAMDA 2018 papers. We will definitely include more datasets in our future works, and we commented on that in the conclusions.

#### Detailed comments and responses (minor issues)

1. *Page 3, Line 26: the bacteria*
  **Response:** Thank you, corrected.

#### Second submission

*The paper has improved much with the changes introduced by the authors, there are some minor issues left with regard to typos and flow of the text.*

#### Minor issues

- *Page 2, Line 12, right: There is a full stop missing - Forensics Challenge. We demonstrate that...*
- *Page 3, Line 19/20, right: “the” is missing - with “the” human reference genome*
- *Page 3, Line 45-52, left: The part would read more fluent if split in two sentences.*
- *Page 3, Line 52/53, left: “reverse complement” instead of reversed complement.*
- *Page 6, Line 26/27, right: “read level”, instead of reads level*

**Response:** Thank you very much for these detailed remarks. We have corrected all these issues.

### Reviewer 3, Chengsheng Zhu

*In this paper, the authors adopted a k-mer comparison-based algorithm that directly assigns metagenomic reads to a group of reference sequences (class). The reference sequences do not have to be taxonomically or functionally annotated – in fact they can be from other metagenomes, which allows circumvention of our limited knowledge of the entire microbial world and makes full use of novel sequences in metagenomes. The authors analyzed MetaSUB dataset from this year’s CAMDA challenge, determined the city origin of unknown samples, and clustered unknown samples of the same origin into the same group. I like the method the authors propose, but have some concerns with how it is presented in the manuscript. My comments are listed below.*

#### Major issues

1. *The methods part lacks important details at several places. For example, the authors attribute each read to the class with the largest match rate score – is there a cutoff below which the read stays unassigned? A read can be assigned to multiple classes if they “have very similar match results” – what is the definition of “very similar”? There is also a final step where the read assignments are analyzed to classify the samples – but the details are completely missing. I would suggest the authors to add the info (or according citations) to the manuscript so that the readers can better understand the method.*
  **Response:** Thank you very much for these comments. We have reviewed the description of our method, as well as we introduced some changes to the method itself (see “Data classification” section), and we have defined exact classification criteria to remove ambiguities. We introduce two thresholds ($\mathbb {T}$ and $\mathbb {M}$) and we analyse the method’s sensitivity to them. This eliminated vague statements like “very similar”.
2. *I have concerns with the authors’ data preprocessing step: the authors concatenate two reads from the same pair with “NN” in between as separators. First of all, N is an alphabet used in sequencing. There could be “NN”s in the original reads, which can cause confusion with the artificially introduced “NN”s. I am more worrisome when it comes to k-mer profiling. The concatenated outputs are now continuous “read”s with always two unknown residues in the middle, while in reality these unknown gaps between the forward and reverse fragments on genomes can vary across different sequencing platforms, usually with sizes much large than two. In my understanding of what the authors did based on the manuscript, they will inevitably generate a large amount of false k-mers, spanning the forward read, the “NN”, and the reverse read, e.g., “XXXXNNXXXX”. These k-mers do not exist in the original metagenomes. Due to the gap length variation in reality, I also doubt the reliability of these k-mers as consistent patterns that fingerprint classes across different sequencing batches. After all, I am not clear of the purpose of this preprocessing step. I don’t intuitively see how the k-mer analysis from the concatenated “read”s is much faster than from the raw reads, in the overall computation time. In fact it generates a lot more k-mers, which are, as discussed above, false signals. If these issues have been taken care of and the preprocessing step is indeed necessary, the authors need to make it clear in the manuscript; otherwise I would suggest to have this step removed.*
  **Response:** Thank you, this description was confusing, indeed. In fact, this is an implementation detail which results from the specific properties of the CoMeta program that exploits KMC. As databases do not contain *k*-mers with ’N’ symbols (this is a feature of KMC), the ’N’ symbols can be added to the query sequence without taking the risk of producing false *k*-mers from the query sequence (the *k*-mers with ’N’s would not be matched with anything in the database). Also, as it is sufficient to use a single ’N’ as a separator, we have changed that to avoid confusion. We have clarified that description in the paper—even though this is an implementation detail, it may be relevant for those who want to reproduce our method (or employ CoMeta for a similar task).
3. *In basic experiments, the authors attempted to account for the imbalanced nature of the data – they removed, from the six largest classes (i.e., city-surface combinations), the k-mers that appeared only once, leaving the rest classes, however, still containing the rare k-mers. I don’t agree with this method as it introduces inconsistency between the top six classes (also why six?) vs. the rest classes. Later in extended experiments, the authors removed rare k-mers from all classes, but this time they didn’t account for the still imbalanced data. I would suggest if the authors were to remove the rare k-mers, this procedure should be carried out to all the classes in both basic and extended experiments. Balancing data can be achieved via, for example, randomly selecting x samples from each class in a bootstrap manner. And data balancing should be carried out in both cases too, unless the authors provide evidence for not to do so.*
  **Response:** In the original manuscript, we included our initial results presented at CAMDA 2018. We agree that these experiments were not performed in a systematic way, so we are not presenting them any more in the revised paper to avoid confusion.
  In the initial studies (presented at CAMDA 2018), this analysis consisted of two steps. In the first one, each read was attributed to that class, whose *Ξ* was the largest. Also, a read could be assigned to several classes, if they had very similar match results, i.e., the subsequent *Ξ* values were greater than 90% of the highest one. Each analyzed *Ξ* had to be greater than or equal to a certain threshold (set to 30%). In the second step, the total number of reads classified to each class was summed for the whole query sample, and it was classified to the class, for which this summed value was the largest. In the extended studies, reported in the paper, we combine the above-mentioned steps and thoroughly examine the impact of various factors on the obtained results.

#### Minor issues

1. *I would suggest the authors to report their performance in actual numbers in additional to listing the tables, e.g., XX% accuracy. This could give the readers a quick and clear impression of the power of their method.*
  **Response:** Thank you, we report the classification accuracy in (%), and we compare the results with other methods.
2. *In the basic experiments for C2 set, the authors only analyzed the first four samples. Are there any specific reasons why not to include all the samples?*
  **Response:** As already mentioned in the response, we showed the results underpinning our initial submission to CAMDA 2018, which were incomplete. We removed that from the paper.
3. *Page 5, line 14: “...We can notice that for three samples (C1_3, C1_14, and C1_21), the classification result has been improved after using k-mer databases without human fragments and infrequent k-mers...” This is not necessarily correct. The authors drew this conclusion from the comparison of C1 sample assignments between the basic (N=23) and extended (N=8) experiments in Table* 7. *One could argue that the driving force for improvements here is the different classes (23 city-surface combinations vs. 8 cities) rather than whether to remove the human DNA and infrequent k-mers. In order to thoroughly assess the effect of human DNA and infrequent k-mers, the authors need to provide assigments comparisons based on the same classes (e.g. N=8) with or without human DNA and infrequent k-mers* (*like in Table* 6). *In fact, Table* 7*showed that further removing more rare k-mers (ci=2 vs ci=4 when N=8) didn’t affect the assignments.*
  **Response:** Thank you for this remark. In the revised manuscript, we report the results only after removing human fragments from the analysis (which is considered a standard procedure).

### Reviewer 4, Andre Kahles (second submission)

*The authors describe new features of CoMeta using a case study based on environmental metagenome samples published in context of the CAMDA 2018 conference. The central theme of the manuscript is to evaluate new features of the previously presented CoMeta method for the (re)-identification/class assignment of metagenome sequence samples. The core strategy is to use the fast kmer counter KMC to generate a sample-specific kmer database. Depending on the application of several levels of filtering and the join of several sample databases into class-level database, a set of reference databases is created that is then used for comparison against the kmer database of the sample to be classified. Based on the text of the manuscript as well as the responses to the previous three reviewers that were made available with the submission, I acknowledge that the text has been streamlined and now comprehensively, though not succinctly, describes motivation, method and evaluation. In general, I believe that the method is an interesting contribution to the pool of tools assessing the similarity of metagenome samples. However, it yet remains to be determined how it would compare against its closest competitors when evaluated in a rigorous manner. The latter is, unfortunately, the strongest weakness of the work. I will summarize my points of criticism below.*

#### Major issues

1. *The work is presented as a case study in context of the CAMDA 2018 conference. As a case study alone, the contributed novelty is limited as the data is not original any more. This leaves a contribution on the methodological side, which requires comparison to other methods, if existing. The authors chose to compare against the results obtained by other submitters to the CAMDA conference. The evaluation presented in the paper includes data revealed at the conference, such as the labels of sample sets C2 and C3. From the text I believe, that no such information was utilized for training in any of the experiments. However, as the results from other submitters to the CAMDA conference are used as a point of comparison, that all did not have access to this data, said comparison can only be a weak point of support. It would be good, if at least one of the competitor’s methods (for instance the one reporting the strongest performance in their contribution) would be run by the authors on the same data and evaluated rigorously. This would allow the reader to see whether the new features of CoMeta increase performance and if so, which one does so the most.*
  **Response:** Thank you very much for this remark. Actually, the primary set was entirely published before the CAMDA challenge, and no information on that set was added afterwards, hence we believe that the comparison for that set is rigorous and it meets all the scientific standards. There have been some differences between the competitors in the adopted evaluation methodology (including leave-one-out and 10-fold cross validation)—we decided to follow the leave-one-out approach, as the results for 10-fold cross validation may depend on how the data are split into the folds. The results obtained using leave-one-out cross validation can be compared between themselves, as there is no randomness involved. For clarity, we have added the following remark in “Results” section:
  It is worth noting that our validation methodology for the primary dataset is identical to that adopted by Zhu [25], and no additional data were published after the CAMDA 2018 competition concerning the primary dataset.
  We agree that the best way for experimental evaluation would be to implement the methods published by other competitors (or used their published implementations), but while the extended abstracts published after CAMDA contain the results that we quote in our paper, they are not detailed enough to reproduce the methods rigorously. We hope that the competitors will also publish full papers with the results they have obtained for all the CAMDA datasets, which would make it possible to compare the methods for the remaining datasets (C1, C2, and C3) as well. Overall, we expect that adopting the leave-one-out approach should help achieve that goal.
2. *The main task to be solved is to determine distances between metagenome samples. There are other methods in the field that require little overhead to run that approximate such distances (such as MASH by Ondov et al.). It would be good to see how CoMeta, which is based on the full kmer spectrum, would compare to such sketching methods.*
  **Response:** Thank you for this suggestion. We decided to implement another variant of our method, in which we use Mash instead of CoMeta for determining the similarity between the samples. We have evaluated that variant for the primary dataset and for C1, as classification accuracy can be evaluated for these cases. The results obtained using Mash instead of CoMeta for the primary dataset are worse than those obtained using other methods, and they are extremely poor for the C1 test set. Overall, the new results that we report and discuss in the revised paper clearly show that our approach is highly sensitive to the tool used for measuring the sample similarity, and that the approximate matching methods like Mash cannot be straightforwardly applied here. Nevertheless, this poses an interesting direction for future work.
3. *The step of hyper parameter tuning is not quite clear to me. From the text I get that the authors use leave-one-out-cross-validation on the 311 samples of the primary dataset to determine values for M and T* (*see Tables* 3*and* 4). *Why is this repeated on the test set C1* (*Tables* 5*and* 6)? *Are both the primary dataset and the C1 set used for fixing the hyper parameters? If yes, how are the results combined?*
  **Response:** Thank you, this was not clearly stated in the paper, indeed. We used the primary set to tune the hyper-parameters. We have repeated the same tests for C1 in order to verify whether the observations made for the primary set are correct for other data. We have commented that in the revised paper.
4. *Some expressions used in the text are not properly defined, e.g., the “match rate score” (page 4). It would be helpful to see how it is computed.*
  **Response:** In the revised paper, we have briefly explained how these expression are computed, and we added a comment that exact algorithm description can be found in our earlier paper [26] (which is published Open Access).

#### Minor issues

1. *I think it would help the understanding of the setup of the classification if Fig.* 2*gets extended to also include the generation of data sets* *D*_1_,...,*D*_*N*_, *including the initial datasets*$D^{0}_{1},..., D^{0}_{N}$*and the removal of human read set* *D*_*H*_.
  **Response:** Thank you for this suggestion—we have extended the figure, and now it includes the step of removing human reads.
2. *In the background the authors write about “unsupervised classification”. This is a bit confusing, as classification is usually a representative of supervised learning. Is it clustering that is actually meant here?*
  **Response:** Yes, we meant “clustering” when using the term “unsupervised classification”. We have clarified that in the text to avoid confusion.
3. *Also in the background the authors refer to “microbiome fingerprints”. This term is not defined and it is not quite clear what exactly the authors mean by this.*
  **Response:** Thank you for drawing our attention to this. Actually, different concepts may be hidden behind this term, so we have clearly defined its meaning in the context of our research:
  Taking that into account, in our work, the microbiome fingerprint is defined as a set of DNA fragments (*k*-mers) derived from organisms living in a given city.
4. *In the section on Data processing (page 3) the authors write “by counting the number of the nucleotides in those k-mers which occur both in the read and in the group”. This is not quite clear to me. The text describes the use of KMC, a kmer counter, but here the authors refer to counting nucleotides.*
  **Response:** In the revised paper, we have added a brief explanation on how the match rate score is computed, but for the details, it is better to refer to our earlier paper [26], in which this is explained thoroughly.
5. *On page 4 the authors write “For each ith read, we create a list of match rate scores...”. This directly follows the description of Fig.* 2, *where the match rate scores are actually grouped by* *D*_*j*_, *rather than by* *R*_*i*_. *Maybe the depiction in Fig.* 2*could be improved?*
  **Response:** Thank you for spotting that—we have corrected that in Fig. 2.
6. *In the current setting, the classification of a dataset follows the “winner takes it all” principle, as this reflects the setup of the experiments. However, it would be interesting if the authors could discuss how robust this is in their evaluations and also comment on other strategies to derive the class label, e.g., through mixing the similarity vectors of all samples of the same group in C2.*
  **Response:** Thank you for this remark. In fact, the samples in the C2 set originate from other places that those covered by the primary set (and this was clearly stated before CAMDA), so there is no reason to combine the partial results (obtained for every sample) at the group level. We agree that it would be an interesting direction for future research to investigate different strategies towards fusing the results obtained from multiple samples, however the dataset would have to be structured in a different way to verify the robustness of such approaches.
7. *Sometimes statements would benefit from some context/interpretation. For instance, in the discussion the authors write: “sample-level filtering of infrequent k-mers is definitely better than if the databases are filtered at the class level”. While this observation is true, it would be interesting to understand why this is the case. Also further down the authors write: “An important advantage of our approach is that... we do not perform taxonomic or functional classification.” Why is this an advantage?*
  **Response:** Thank you for these questions. We have tried to clarify that in the revised paper. Regarding filtering the infrequent *k*-mers, we have added the following comment:
  Probably this is due to the fact that during the sample-level filtering, only these *k*-mers are selected, which occur at least a few times in a single sample (which reduces the risk of selecting *k*-mers present due to sequencing errors). During the class-level filtering, single erroneous *k*-mers can be accumulated, as the databases at the class level are much larger. Possibly, the *ci* parameter should depend on the database size, but this would have to be verified.
  Regarding avoiding taxonomic or functional classification, the advantages are twofold: (i) we do not need large reference databases to perform the analysis, and (ii) we may exploit even these fragments which are not covered by existing databases (they do not cover all of the existing organisms). We have commented that in “Discussion” section.
8. *In the Conclusion the authors write about “open-world classification”. What is meant by this?*
  **Response:** We use this term in “Discussion” section for the first time, hence we have added an explanation there:
  ...it would be difficult to establish a cut-off threshold required for open-world classification (when it is unknown whether a sample originates from the places covered by the reference set).
9. *The authors are tuning hyper parameters M and T but not k. Why?*
  **Response:** The influence of the hyper-parameter *k* has been deeply analysed in the literature, so we have focused on the new hyper-parameters in our research, assuming sensible value of *k*. We are planning to verify whether *k* (as well as *ci*) can be better tuned, but this would require much larger computational effort. This problem is commented in Conclusions:
  Also, it could be explored more deeply how the preprocessing affects the classification accuracy—this may include checking the influence of removing human DNA or filtering infrequent *k*-mers using different values of *ci*, as well as tuning the value of *k* (i.e., the length of *k*-mers).

#### Some grammar issues/typos

1. *page 2: We demonstrate that it is not necessary to identify neither the organisms, nor their functions*... →*We demonstrate that it is not necessary to identify the organisms or their functions...*
2. *page 3: The majority of studies on metagenome... → The majority of studies on metagenomes...*
3. *page 3:... allows a database being built*... →... *allows for building a database...*
4. *page 5: sensitiveness (used twice)* →*sensitivity*
5. *page 6:... with the accuracy of*... →*with an accuracy of...*
  **Response:** Thank you for these detailed remarks—we have corrected the paper following your comments.

## Supplementary information

**Additional file 1** The file includes specification of all the samples in the primary dataset.

## Abbreviations

*A**C**C*: overall classification accuracy
AKL: New Zealand, Auckland
*C*1: first test set
*C*2: second test set
*C*3: third test set
*c**i*=*x*: a parameter of the KMC_tools software, which excludes *k*-mers occurring less than *x* times in the *k*-mer database
*D*_*H*_: *k*-mer database for the human reference sequence
*D*_*i*_: *i*th *k*-mer database
*F**P*: the number of samples incorrectly labeled as belonging to that *i*th class
HAM: New Zealand, Hamilton
*k*-mers: all substrings in the sequence of the length of *k*
$\mathbb {M}$: maximum class number threshold
*N*: number of classes
NYC: USA, New York
OFA: Nigeria, Offa
*P**P**V*: precision (positive predictive value)
PXO: Portugal, Porto
*R*_*i*_: *i*th read
SAC: USA, Sacramento
SCL: Chile, Santiago
$\mathbb {T}$: absolute threshold in proposed classification process
TOK: Japan, Tokyo
*T**P*: the number of correctly classified samples
*T**P**R*: recall (true positive rate)
*Ξ*_*RiDj*_: the result of a single matching for *i*th class and *j*th read (*match rate score*)
