# Supplementary material for: Environmental metagenome classification for constructing a microbiome fingerprint
Source: Biol Direct. 2019 Nov 13;14:20. doi: 10.1186/s13062-019-0251-z (PMC6854650; doi:10.1186/s13062-019-0251-z)
Supplement: Supplementary file 1 — Additional file 1 The file includes specification of all the samples in the primary dataset. [file 13062_2019_251_MOESM1_ESM.pdf]

Table 1: **Specification of all the samples in the primary dataset.** The 2nd column shows the number of reads in the original samples. If the sample contains paired-end reads (see the 5th column), then the actual number of the reads is twice as large. The 3rd column indicates the number of the reads after removing human DNA fragments. The 6th column includes the total number of separate k-mers ($k = 24$) within the reads in the sample. The next three columns (7th–9th) include the number of unique k-mers before and after removing human DNA fragments. The databases are subsequently filtered to reject the k-mers which appear less than ci times, as they may result from sequencing errors.

Sample ID	Number of reads in sample		Avg. read length	Paired-end seq.	Total number of 24-mers	Number of unique 24-mers in the k-mer database, $ci = 1$	Number of unique 24-mers in the k-mer database (noHum), $ci = 1$	Number of unique 24-mers in the k-mer database (noHum), $ci = 4$
	Original data	Without human DNA (noHum)						
AKL-001	3 737 675	3 668 857	151	yes	956 800 028	143 090 617	142 943 258	31 952 388
AKL-002	5 422 649	5 350 169	151	yes	1 388 133 229	100 109 059	97 233 683	15 053 555
AKL-003	5 191 719	5 142 454	151	yes	1 329 013 667	150 270 399	150 092 417	42 467 952
AKL-004	4 209 429	4 166 956	151	yes	1 077 561 188	168 178 983	167 621 524	50 623 562
AKL-005	5 893 643	5 798 481	151	yes	1 508 698 874	129 081 960	128 935 860	25 761 393
AKL-006	5 824 972	5 799 817	151	yes	1 491 125 937	143 364 217	143 273 613	40 841 414
AKL-007	4 561 451	4 517 541	151	yes	1 167 679 628	164 404 117	164 242 639	29 364 291
AKL-008	5 138 623	5 108 608	151	yes	1 315 422 758	128 588 695	128 484 511	29 043 410
AKL-009	4 417 644	4 317 259	151	yes	1 130 860 651	211 978 125	211 850 200	61 831 150
AKL-010	3 955 447	3 872 917	151	yes	1 012 544 711	133 475 468	133 377 875	43 582 781
AKL-011	4 693 836	4 597 106	151	yes	1 201 565 120	213 869 584	213 739 340	69 297 127
AKL-012	6 225 730	5 904 874	151	yes	1 593 711 434	688 009 879	679 911 030	77 749 699
AKL-013	5 459 799	5 365 320	151	yes	1 397 634 797	124 236 018	124 061 912	20 055 495
AKL-014	3 756 041	3 719 059	151	yes	961 498 343	227 607 785	227 527 539	66 037 025
AKL-015	5 453 802	5 416 242	151	yes	1 396 105 060	168 335 529	168 131 280	42 429 578
HAM-001	5 752 951	5 718 008	151	yes	1 472 684 993	211 011 721	210 861 892	69 526 770
HAM-002	5 784 122	5 704 706	151	yes	1 480 662 628	354 110 888	353 959 329	92 290 328
HAM-003	6 145 807	6 105 201	151	yes	1 573 248 200	268 734 207	268 582 656	48 744 914
HAM-004	9 590 761	9 498 487	151	yes	2 455 115 234	577 272 428	577 051 928	191 372 436
HAM-005	6 711 271	6 635 970	151	yes	1 718 000 321	509 517 959	509 323 755	109 822 795
HAM-006	5 206 924	5 153 997	151	yes	1 332 909 244	164 653 202	164 527 907	49 871 738
HAM-007	9 015 378	8 964 367	151	yes	2 307 823 738	289 310 709	289 115 562	88 416 568
HAM-008	3 080 193	2 953 439	151	yes	788 488 627	248 315 648	248 076 246	37 612 775

Continued on the next page...

Table 1—continued

Sample ID	Original data	Without human DNA (noHum)	Avg. read length	Paired-end seq.	Total number of 24-mers	Number of unique 24-mers in the k-mer database, $ci = 1$	Number of unique 24-mers in the k-mer database (noHum), $ci = 1$	Number of unique 24-mers in the k-mer database (noHum), $ci = 4$
HAM-009	6 031 377	5 959 584	151	yes	1 543 961 874	229 992 912	229 837 139	71 063 899
HAM-010	5 407 668	5 349 449	151	yes	1 384 302 817	206 006 192	205 810 211	78 577 387
HAM-011	5 085 516	4 993 335	151	yes	1 301 828 317	178 730 066	178 346 120	61 294 378
HAM-012	4 528 997	4 483 176	151	yes	1 159 363 561	317 020 253	316 861 086	79 034 629
HAM-013	9 483 142	9 423 838	151	yes	2 427 569 403	165 335 010	165 111 633	17 263 026
HAM-014	6 026 425	5 804 651	151	yes	1 542 692 316	255 442 123	242 010 795	67 539 732
HAM-015	5 583 483	5 561 099	151	yes	1 429 301 636	111 601 866	111 478 060	16 215 392
HAM-016	3 746 375	3 686 075	151	yes	959 030 151	220 361 639	220 095 026	37 301 174
NYC-001	17 224 094	12 430 542	151	yes	4 407 476 028	2 311 523 539	2 077 384 688	126 628 031
NYC-002	13 042 332	7 998 131	151	yes	3 337 417 116	1 357 634 389	1 107 568 742	69 663 812
NYC-003	19 684 314	13 016 172	151	yes	5 037 044 610	1 632 584 528	1 333 166 695	94 848 516
NYC-004	17 201 082	12 955 158	151	yes	4 401 576 609	2 307 860 396	2 118 891 789	135 878 732
NYC-005	34 611 323	19 148 172	151	yes	8 856 735 827	4 430 622 249	3 782 114 673	246 474 268
NYC-006	31 266 281	10 373 455	151	yes	8 000 958 616	3 827 331 190	2 925 086 949	218 154 946
NYC-007	23 918 726	20 480 890	151	yes	6 120 125 312	1 921 148 530	1 782 934 400	285 335 814
NYC-008	23 725 498	12 060 793	151	yes	6 070 684 306	3 400 217 471	2 797 053 364	154 698 757
NYC-009	23 672 991	17 933 233	151	yes	6 057 812 738	3 187 492 191	2 985 595 040	187 954 373
NYC-010	23 738 464	13 369 010	151	yes	6 075 275 268	3 208 782 375	2 744 886 454	171 547 178
NYC-011	23 076 691	21 642 818	151	yes	5 905 126 556	1 435 060 436	1 374 013 607	194 253 731
NYC-012	23 737 683	12 874 023	151	yes	6 074 187 550	2 903 484 332	2 410 979 305	179 478 537
NYC-013	20 124 237	17 462 858	151	yes	5 149 626 124	2 007 445 933	1 898 358 419	157 960 862
NYC-014	24 755 713	21 443 002	151	yes	6 334 734 868	3 552 589 462	3 432 732 144	190 761 261
NYC-015	15 934 869	14 835 686	151	yes	4 077 567 459	962 295 668	921 496 310	103 875 395
NYC-016	29 995 923	22 468 885	151	yes	7 675 709 257	4 122 433 347	3 830 537 678	250 181 847
NYC-017	21 680 792	14 998 983	151	yes	5 547 913 286	3 052 107 582	2 771 368 572	156 509 471
NYC-018	24 596 752	18 078 540	151	yes	6 294 075 546	2 524 098 020	2 216 082 787	157 842 145
NYC-019	21 891 303	12 930 191	151	yes	5 601 788 944	2 936 698 928	2 519 248 900	158 224 185
NYC-020	18 928 348	15 564 877	151	yes	4 843 606 016	2 306 165 801	2 149 418 295	147 329 199
NYC-021	28 504 847	25 484 905	151	yes	7 294 130 389	2 495 834 578	2 380 100 434	194 584 275
NYC-022	21 709 981	15 913 376	151	yes	5 555 409 453	3 045 287 048	2 793 056 496	175 153 108
NYC-023	30 988 066	24 957 604	151	yes	7 929 590 511	3 161 975 080	2 873 334 161	205 895 606
NYC-024	18 240 047	16 717 188	151	yes	4 667 449 523	1 141 113 570	1 069 308 104	129 796 394
NYC-025	18 949 456	13 154 165	151	yes	4 849 012 584	2 661 506 792	2 399 695 483	138 843 811
NYC-026	18 676 859	13 709 733	151	yes	4 779 271 390	2 587 982 991	2 361 651 735	155 689 771

Continued on the next page...

Table 1—continued

Sample ID	Original data	Without human DNA (noHum)	Avg. read length	Paired-end seq.	Total number of 24-mers	Number of unique 24-mers in the k-mer database, $ci = 1$	Number of unique 24-mers in the k-mer database (noHum), $ci = 1$	Number of unique 24-mers in the k-mer database (noHum), $ci = 4$
NYC-027	7 817 978	7 730 062	101	yes	1 206 527 532	219 427 195	219 335 129	27 200 476
NYC-028	4 474 744	4 438 630	125	yes	911 927 021	165 573 068	165 533 122	34 214 898
NYC-029	5 698 506	5 617 475	101	yes	879 361 975	260 179 083	260 097 211	52 370 669
NYC-030	4 420 446	4 354 212	125	yes	900 828 818	233 813 501	233 724 217	63 658 394
NYC-031	4 211 255	4 188 610	125	yes	858 249 321	116 058 785	116 030 523	16 826 659
NYC-032	3 340 705	3 305 821	125	yes	680 831 323	178 052 348	177 979 385	46 304 602
NYC-033	4 369 600	4 300 088	101	yes	674 255 428	145 562 301	145 495 059	38 988 608
NYC-034	4 405 631	4 376 258	125	yes	897 854 808	158 491 743	158 439 223	25 925 176
NYC-035	5 582 819	5 426 056	101	yes	861 372 729	129 628 849	129 529 172	19 596 131
NYC-036	4 130 181	4 088 136	125	yes	841 722 411	233 041 898	232 946 503	43 696 851
NYC-037	3 740 837	3 684 031	125	yes	762 236 853	113 703 342	113 678 254	11 431 759
NYC-038	4 102 587	4 076 441	125	yes	836 064 941	154 393 359	154 319 681	30 424 180
NYC-039	5 692 408	5 557 415	101	yes	878 399 577	249 133 097	248 747 280	59 985 601
NYC-040	3 026 495	2 986 147	125	yes	616 805 588	180 018 764	179 937 623	32 423 336
NYC-041	3 968 809	3 928 383	125	yes	808 827 065	204 254 951	204 186 787	35 936 198
NYC-042	7 072 158	6 977 792	101	yes	1 091 347 432	214 221 054	214 124 607	54 561 967
NYC-043	3 867 695	3 837 044	125	yes	788 255 097	117 820 970	117 765 380	15 425 913
NYC-044	8 427 605	8 291 172	101	yes	1 300 562 172	322 296 913	322 149 645	70 781 320
NYC-045	2 878 465	2 863 116	125	yes	586 668 069	114 040 399	114 015 121	18 524 372
NYC-046	3 504 938	3 478 948	125	yes	714 184 424	142 574 115	142 529 511	18 590 806
NYC-047	6 883 193	6 817 597	101	yes	1 062 300 990	199 961 791	199 889 666	39 437 971
NYC-048	4 056 101	4 024 378	125	yes	826 578 508	173 465 524	173 397 611	26 589 282
NYC-049	4 052 551	4 023 826	125	yes	825 900 498	122 763 211	122 745 714	11 319 632
NYC-050	3 448 847	3 416 437	125	yes	702 810 934	165 829 347	165 786 037	31 601 564
NYC-051	3 868 346	3 846 485	125	yes	788 394 009	106 388 966	106 358 412	9 656 379
NYC-052	4 717 513	4 679 929	125	yes	961 396 056	208 321 717	208 270 095	43 678 515
NYC-053	7 405 044	7 328 894	101	yes	1 142 679 509	198 242 681	198 122 775	23 372 225
NYC-054	6 833 000	6 767 753	101	yes	1 054 504 192	218 677 491	218 598 697	42 782 851
NYC-055	7 379 235	7 294 865	101	yes	1 138 785 440	225 476 348	225 384 578	48 621 802
NYC-056	2 650 628	2 638 844	125	yes	540 235 264	114 636 754	114 621 892	35 932 810
NYC-057	5 010 168	4 950 228	101	yes	773 197 303	151 544 568	151 468 696	35 413 935
NYC-058	6 558 656	6 481 069	101	yes	1 012 170 700	236 227 449	236 082 044	47 091 955
NYC-059	5 578 578	5 515 475	101	yes	860 951 432	248 176 690	248 101 886	46 011 729
NYC-060	3 097 955	3 067 823	125	yes	631 330 925	197 005 280	196 953 407	31 609 450

Continued on the next page...

Table 1—continued

Sample ID	Original data	Without human DNA (noHum)	Avg. read length	Paired-end seq.	Total number of 24-mers	Number of unique 24-mers in the k-mer database, $ci = 1$	Number of unique 24-mers in the k-mer database (noHum), $ci = 1$	Number of unique 24-mers in the k-mer database (noHum), $ci = 4$
NYC-061	5 149 182	5 086 486	101	yes	794 584 600	223 596 446	223 526 872	38 992 044
NYC-062	2 154 116	2 130 988	125	yes	439 016 600	122 579 720	122 513 604	31 675 384
NYC-063	6 177 448	6 124 166	101	yes	954 956 181	141 833 147	141 777 450	16 021 257
NYC-064	2 268 883	2 227 832	125	yes	462 351 573	171 705 023	171 625 451	33 178 157
NYC-065	3 466 465	3 432 544	125	yes	706 486 219	166 705 800	166 674 053	47 688 903
NYC-066	5 783 549	5 671 239	101	yes	892 537 761	211 141 681	211 040 483	51 426 144
NYC-067	5 635 983	5 583 275	101	yes	869 847 597	190 071 290	189 979 874	28 995 066
NYC-068	2 796 929	2 778 109	125	yes	570 042 598	136 040 410	135 989 044	24 095 798
NYC-069	3 324 710	3 305 824	125	yes	677 505 554	132 073 721	132 036 592	24 853 503
NYC-070	3 092 030	3 072 506	125	yes	630 183 845	121 693 435	121 671 088	24 870 862
NYC-071	3 223 598	3 194 853	125	yes	656 868 269	146 208 785	145 619 786	33 597 016
NYC-072	3 255 181	3 236 922	125	yes	663 400 896	117 812 072	117 782 485	18 722 044
NYC-073	2 733 259	2 714 756	125	yes	557 020 944	129 773 849	129 741 965	27 421 574
NYC-074	8 551 873	8 308 564	101	yes	1 319 687 856	252 720 432	252 560 529	50 979 174
NYC-075	3 610 952	3 583 227	125	yes	735 925 344	180 529 699	180 476 608	38 413 919
NYC-076	5 756 863	5 695 249	101	yes	888 580 037	182 193 182	182 136 019	35 449 989
NYC-077	2 320 375	2 306 355	125	yes	472 864 423	65 738 049	65 721 709	12 729 145
NYC-078	5 920 644	5 855 597	101	yes	915 207 391	173 234 321	173 168 504	40 428 244
NYC-079	807 478	793 405	300	yes	447 654 589	120 042 079	120 026 811	23 241 189
NYC-080	2 905 795	2 894 923	125	yes	592 208 141	103 867 156	103 834 891	20 757 249
NYC-081	3 290 122	3 267 138	125	yes	670 533 089	145 795 114	145 493 314	32 579 649
NYC-082	2 988 469	2 923 793	125	yes	608 924 303	171 827 810	171 688 874	42 178 759
NYC-083	3 338 754	3 257 852	125	yes	680 425 087	207 448 310	203 848 746	37 849 033
NYC-084	8 276 691	8 100 596	101	yes	1 277 222 783	231 331 128	231 147 618	52 355 459
NYC-085	6 146 942	6 097 164	101	yes	948 741 523	156 516 201	156 471 755	21 855 208
NYC-086	3 608 437	3 571 180	125	yes	735 336 605	148 606 352	148 559 807	35 630 171
NYC-087	3 447 661	3 429 888	125	yes	702 604 378	142 823 934	142 785 019	26 394 665
NYC-088	5 518 627	5 457 612	101	yes	851 743 794	165 212 488	165 151 297	28 906 800
NYC-089	1 194 877	1 184 567	125	yes	243 529 002	77 218 534	77 172 769	17 257 800
NYC-090	3 549 075	3 532 311	125	yes	723 339 403	97 825 290	97 747 607	9 509 513
NYC-091	3 130 199	3 112 437	125	yes	637 982 692	124 581 629	124 546 975	21 652 067
NYC-092	3 810 764	3 784 883	125	yes	776 592 274	130 065 140	130 046 906	13 593 514
NYC-093	6 307 804	6 254 391	101	yes	973 597 294	179 933 439	179 872 548	23 857 244
NYC-094	9 003 296	8 910 778	101	yes	1 389 539 273	320 988 887	320 847 911	43 160 865

Continued on the next page...

Table 1—continued

Sample ID	Original data	Without human DNA (noHum)	Avg. read length	Paired-end seq.	Total number of 24-mers	Number of unique 24-mers in the k-mer database, $ci = 1$	Number of unique 24-mers in the k-mer database (noHum), $ci = 1$	Number of unique 24-mers in the k-mer database (noHum), $ci = 4$
NYC-095	3 859 885	3 839 070	125	yes	786 620 697	114 591 162	114 546 804	18 322 768
NYC-096	3 090 785	3 071 152	125	yes	629 943 700	121 933 948	121 894 219	27 026 777
NYC-097	2 859 778	2 819 806	125	yes	582 815 605	171 601 317	170 403 077	40 984 633
NYC-098	2 379 710	2 358 040	125	yes	484 981 421	80 817 996	80 732 435	17 346 370
NYC-099	3 229 617	3 204 925	125	yes	658 225 308	125 838 506	125 672 997	16 958 125
NYC-100	6 401 468	6 319 115	101	yes	988 006 013	218 454 885	218 389 270	49 025 821
NYC-101	2 165 303	2 087 923	125	yes	441 207 530	92 546 569	92 396 228	23 554 303
NYC-102	9 167 728	9 084 702	101	yes	1 415 027 179	251 818 839	251 728 962	34 961 256
NYC-103	7 133 688	7 056 607	101	yes	1 100 990 763	237 962 842	237 879 509	37 864 141
NYC-104	9 964 337	9 817 915	101	yes	1 537 770 044	297 507 288	297 164 353	58 563 788
NYC-105	2 617 911	2 594 976	125	yes	533 542 395	96 898 815	96 736 601	10 695 887
NYC-106	3 498 941	3 483 906	125	yes	713 081 647	120 953 322	120 927 046	27 747 968
NYC-107	8 982 559	8 897 794	101	yes	1 386 347 516	243 856 990	243 774 058	41 079 383
NYC-108	7 988 909	7 898 127	101	yes	1 232 978 245	223 937 181	223 860 475	36 498 988
NYC-109	3 103 510	3 071 114	125	yes	632 435 699	161 257 425	161 211 984	31 354 748
NYC-110	2 423 178	2 404 697	125	yes	493 826 757	125 241 222	125 206 684	15 511 640
NYC-111	7 831 651	7 632 326	101	yes	1 208 464 850	141 396 950	141 235 339	22 170 137
NYC-112	9 327 327	9 225 978	101	yes	1 439 607 896	231 043 748	230 921 353	22 133 238
NYC-113	3 136 999	3 109 745	125	yes	639 313 169	119 571 336	119 456 255	14 511 793
NYC-114	7 032 719	6 935 411	101	yes	1 087 037 291	195 347 081	195 232 771	36 998 170
NYC-115	2 356 841	2 336 032	125	yes	480 340 921	97 634 086	97 489 149	13 896 215
NYC-116	8 551 403	8 425 965	101	yes	1 321 902 858	199 472 479	199 136 628	32 263 875
NYC-117	2 605 650	2 580 955	125	yes	530 938 753	113 232 586	113 198 499	30 904 576
NYC-118	2 365 893	2 334 523	125	yes	482 103 747	121 972 314	121 910 299	20 606 765
NYC-119	3 500 283	3 475 253	125	yes	713 352 668	124 582 495	124 505 782	19 416 986
NYC-120	3 327 579	3 309 052	125	yes	678 230 830	117 363 285	117 303 807	19 063 703
NYC-121	8 549 864	8 462 804	101	yes	1 319 612 879	201 987 616	201 929 156	24 296 368
NYC-122	2 575 302	2 545 563	125	yes	524 845 416	110 299 862	110 217 539	26 470 606
NYC-123	7 486 896	7 426 586	101	yes	1 155 528 975	169 775 824	169 719 649	17 835 273
NYC-124	11 693 538	11 328 957	101	yes	1 804 494 574	306 836 397	306 367 600	52 837 421
NYC-125	1 668 582	1 589 144	125	yes	340 007 180	107 553 259	107 126 122	22 712 394
NYC-126	3 546 147	3 531 138	125	yes	722 714 214	106 536 375	106 513 763	13 848 159
OFA-001	24 759 619	24 254 250	151	yes	6 335 742 508	969 773 957	969 034 084	189 038 082
OFA-002	29 212 709	28 708 511	151	yes	7 475 242 510	1 495 190 663	1 494 200 803	332 108 144

Continued on the next page...

Table 1—continued

Sample ID	Original data	Without human DNA (noHum)	Avg. read length	Paired-end seq.	Total number of 24-mers	Number of unique 24-mers in the k-mer database, $ci = 1$	Number of unique 24-mers in the k-mer database (noHum), $ci = 1$	Number of unique 24-mers in the k-mer database (noHum), $ci = 4$
OFA-003	26 851 260	26 450 961	151	yes	6 871 043 080	1 126 994 483	1 126 213 469	262 920 964
OFA-004	27 529 049	26 938 752	151	yes	7 044 449 249	1 254 154 068	1 252 836 098	263 853 156
OFA-005	45 913 255	44 877 656	151	yes	11 748 814 698	1 536 917 136	1 535 176 165	295 921 338
OFA-006	40 841 192	40 309 410	151	yes	10 450 895 540	1 394 868 399	1 393 606 085	233 951 385
OFA-007	39 916 805	39 183 091	151	yes	10 214 419 160	1 876 677 376	1 875 518 293	445 034 980
OFA-008	32 068 918	31 730 207	151	yes	8 206 168 558	1 677 026 929	1 676 201 328	299 773 701
OFA-009	33 837 863	33 487 187	151	yes	8 658 828 535	1 105 623 393	1 104 633 745	147 772 417
OFA-010	27 000 749	26 426 580	151	yes	6 909 315 147	1 230 092 062	1 229 156 056	256 116 503
OFA-011	43 614 712	43 295 757	151	yes	11 160 688 836	1 098 126 243	1 097 058 736	140 972 942
OFA-012	57 596 787	56 762 620	151	yes	14 738 126 852	2 713 473 130	2 712 034 372	475 072 423
OFA-013	47 453 987	47 035 351	151	yes	12 143 235 650	1 502 191 658	1 501 432 394	148 094 964
OFA-014	29 839 152	29 313 198	151	yes	7 635 567 711	1 478 962 592	1 477 976 723	308 501 408
OFA-015	21 950 913	21 256 943	151	yes	5 616 964 798	871 331 316	870 493 216	195 003 593
OFA-016	50 885 699	50 364 595	151	yes	13 021 097 060	1 552 159 975	1 550 687 988	198 423 801
OFA-017	43 724 144	42 853 478	151	yes	11 188 551 739	1 499 736 311	1 498 203 375	275 440 292
OFA-018	10 005 417	9 832 803	151	yes	2 560 388 875	525 102 107	524 783 931	110 966 867
OFA-019	43 393 242	42 998 161	151	yes	11 104 036 239	1 526 334 166	1 525 291 293	283 728 103
OFA-020	32 998 043	32 644 001	151	yes	8 443 958 387	1 239 583 239	1 238 481 972	236 136 747
PXO-001	4 460 731	3 833 040	151	yes	1 141 571 099	532 238 649	526 303 281	46 827 373
PXO-002	4 446 819	3 495 848	151	yes	1 138 019 181	500 857 830	482 352 743	34 960 298
PXO-003	4 356 374	3 888 285	151	yes	1 114 885 106	505 729 913	501 225 183	41 307 856
PXO-004	4 254 956	3 649 446	151	yes	1 088 933 283	462 780 637	457 968 096	54 084 528
PXO-005	3 727 806	2 598 001	151	yes	954 009 950	397 135 619	370 389 020	33 974 546
PXO-006	4 626 027	3 164 892	151	yes	1 183 879 396	493 345 387	450 051 147	57 596 759
PXO-007	4 233 688	1 287 868	151	yes	1 083 478 375	448 293 968	329 727 529	46 670 778
PXO-008	5 481 447	4 884 807	151	yes	1 402 800 572	635 881 751	631 045 417	78 359 788
PXO-009	6 259 470	4 533 182	151	yes	1 601 929 140	689 747 625	645 778 849	67 632 583
PXO-010	5 255 662	3 684 029	151	yes	1 345 018 893	586 379 722	542 793 628	49 227 243
PXO-011	5 805 125	3 386 119	151	yes	1 485 641 389	645 925 165	559 735 331	46 792 501
PXO-012	7 137 552	5 850 282	151	yes	1 826 633 419	711 244 382	700 430 801	99 808 348
PXO-013	4 455 561	3 580 399	151	yes	1 140 279 429	476 073 041	469 059 577	42 128 227
PXO-014	6 865 265	5 107 252	151	yes	1 756 931 359	761 239 166	713 184 841	90 358 812
PXO-015	4 383 603	2 840 364	151	yes	1 121 839 183	460 292 879	410 901 442	68 232 635
PXO-016	5 195 670	2 485 883	151	yes	1 329 672 163	546 825 770	448 325 829	61 211 959

Continued on the next page...

Table 1—continued

Sample ID	Original data	Without human DNA (noHum)	Avg. read length	Paired-end seq.	Total number of 24-mers	Number of unique 24-mers in the k-mer database, $ci = 1$	Number of unique 24-mers in the k-mer database (noHum), $ci = 1$	Number of unique 24-mers in the k-mer database (noHum), $ci = 4$
PXO-017	4 342 634	3 028 457	151	yes	1 111 353 530	502 556 144	459 847 340	39 091 446
PXO-018	4 605 185	2 469 716	151	yes	1 178 566 923	554 236 101	468 547 981	50 884 699
PXO-019	4 934 448	3 940 823	151	yes	1 262 815 331	539 209 545	527 747 134	69 121 288
PXO-020	4 286 217	2 396 753	151	yes	1 096 907 453	468 550 653	399 682 045	42 496 862
PXO-021	3 127 444	1 933 346	151	yes	800 366 297	317 444 833	285 893 308	42 514 695
PXO-022	4 442 775	2 338 819	151	yes	1 136 992 885	478 899 568	403 254 407	48 162 671
PXO-023	4 133 784	1 356 657	151	yes	1 057 915 149	448 065 198	333 740 621	46 249 523
PXO-024	4 552 286	2 875 861	151	yes	1 165 017 797	480 332 226	430 770 609	61 067 842
PXO-025	5 811 914	4 366 117	151	yes	1 487 380 791	672 738 232	625 626 116	83 666 990
PXO-026	4 629 880	2 748 927	151	yes	1 184 868 976	504 557 878	442 814 812	43 330 252
PXO-027	5 603 769	5 040 587	151	yes	1 434 118 484	574 112 574	570 074 750	79 511 128
PXO-028	5 833 524	4 649 649	151	yes	1 492 897 995	668 191 014	650 678 439	77 647 817
PXO-029	4 985 616	2 339 919	151	yes	1 275 908 839	540 239 891	435 975 428	61 452 701
PXO-030	5 026 138	2 384 002	151	yes	1 286 285 441	522 432 680	426 840 174	62 147 682
PXO-031	4 570 107	3 210 328	151	yes	1 169 574 604	487 377 958	454 339 655	44 130 782
PXO-032	5 339 150	2 389 272	151	yes	1 366 378 731	628 062 431	504 233 111	54 210 496
PXO-033	3 424 319	2 497 988	151	yes	876 362 295	380 851 958	358 019 680	30 033 057
PXO-034	4 198 144	3 619 627	151	yes	1 074 381 761	469 732 854	461 113 961	42 286 563
PXO-035	4 377 582	4 123 133	151	yes	1 120 289 681	458 935 904	457 025 298	73 793 931
PXO-036	4 714 981	2 829 477	151	yes	1 206 666 489	508 209 827	452 010 506	64 027 057
PXO-037	5 651 964	3 658 845	151	yes	1 446 433 593	609 584 703	547 558 004	74 485 716
PXO-038	4 416 484	3 283 599	151	yes	1 130 261 328	427 314 413	419 295 214	66 804 674
PXO-039	4 537 605	2 431 595	151	yes	1 161 275 425	491 240 197	416 598 171	44 727 216
PXO-040	4 183 519	2 677 777	151	yes	1 070 642 463	452 608 611	409 415 598	45 193 095
PXO-041	4 179 080	2 155 032	151	yes	1 069 498 220	465 944 219	389 045 442	40 651 260
PXO-042	4 278 462	1 785 121	151	yes	1 094 941 567	458 766 767	362 452 476	45 865 366
PXO-043	4 393 238	3 477 708	151	yes	1 124 310 608	488 868 116	464 229 839	55 931 496
PXO-044	2 785 354	1 962 355	151	yes	712 824 634	278 901 010	263 907 201	40 733 137
PXO-045	7 374 430	5 379 507	151	yes	1 887 274 034	806 455 449	756 173 466	82 531 789
PXO-046	6 448 166	3 985 262	151	yes	1 650 200 861	743 169 727	649 102 893	75 713 037
PXO-047	7 150 822	5 636 085	151	yes	1 830 032 707	783 822 857	761 203 719	68 477 585
PXO-048	7 904 686	3 883 191	151	yes	2 022 928 642	876 840 767	716 545 424	74 441 728
PXO-049	8 376 606	6 699 857	151	yes	2 143 738 223	1 000 471 322	951 283 826	110 418 535
PXO-050	7 644 580	5 461 178	151	yes	1 956 384 098	857 547 512	773 551 934	112 264 868

Continued on the next page...

Table 1—continued

Sample ID	Original data	Without human DNA (noHum)	Avg. read length	Paired-end seq.	Total number of 24-mers	Number of unique 24-mers in the k-mer database, $ci = 1$	Number of unique 24-mers in the k-mer database (noHum), $ci = 1$	Number of unique 24-mers in the k-mer database (noHum), $ci = 4$
PXO-051	8 368 185	6 216 654	151	yes	2 141 575 227	879 951 044	825 779 026	135 547 778
PXO-052	6 383 048	2 820 587	151	yes	1 633 527 318	683 829 423	548 725 962	67 291 644
PXO-053	4 797 726	2 300 955	151	yes	1 227 820 854	508 197 209	416 460 343	56 074 171
PXO-054	5 404 801	4 163 688	151	yes	1 383 199 851	567 566 967	548 459 392	68 447 980
PXO-055	5 467 411	4 774 235	151	yes	1 399 207 091	489 003 951	485 993 199	104 078 951
PXO-056	3 085 614	904 138	151	yes	789 662 680	342 090 206	249 634 101	32 798 444
PXO-057	5 573 523	4 881 332	151	yes	1 426 382 913	587 459 156	571 277 009	95 869 152
PXO-058	5 029 782	1 483 512	151	yes	1 287 217 754	535 780 661	389 767 642	55 349 237
PXO-059	4 066 683	2 320 500	151	yes	1 040 732 878	429 806 536	372 902 891	45 192 231
PXO-060	4 646 676	3 217 707	151	yes	1 189 180 596	526 799 195	479 891 791	63 838 787
SAC-001	4 140 469	3 996 970	151	yes	1 059 914 117	699 100 179	696 143 349	26 386 996
SAC-002	3 319 135	2 399 018	151	yes	849 654 046	517 461 834	476 976 973	20 667 162
SAC-003	4 798 681	4 619 733	151	yes	1 228 402 651	725 098 706	719 729 481	47 841 664
SAC-004	4 498 581	4 276 997	151	yes	1 151 576 929	734 305 754	732 758 276	29 936 669
SAC-005	4 924 782	4 742 248	151	yes	1 260 681 770	859 428 699	855 112 022	27 559 214
SAC-006	5 096 193	4 752 857	151	yes	1 304 561 107	859 269 399	853 639 311	20 646 823
SAC-007	2 261 456	2 082 472	151	yes	578 902 524	390 958 350	385 409 342	10 669 043
SAC-008	4 123 868	3 911 404	151	yes	1 055 663 371	699 149 500	697 902 604	16 764 029
SAC-009	3 603 047	3 422 910	151	yes	922 336 625	623 145 408	620 639 040	19 557 520
SAC-010	3 732 829	3 512 605	151	yes	955 558 381	650 550 890	648 379 228	18 434 076
SAC-011	3 633 066	3 340 702	151	yes	930 019 613	610 479 178	603 834 702	18 874 129
SAC-012	3 313 170	3 039 460	151	yes	848 129 650	577 301 639	571 956 690	14 982 549
SAC-013	4 575 877	4 181 854	151	yes	1 171 371 020	773 132 361	758 459 996	24 392 958
SAC-014	4 883 684	4 286 897	151	yes	1 250 159 259	756 009 092	743 200 910	25 415 398
SAC-015	3 382 090	2 475 695	151	yes	865 772 990	549 024 234	501 698 081	16 043 986
SAC-016	2 689 672	2 500 687	151	yes	688 522 836	472 059 171	463 346 875	12 556 387
SAC-017	44 579 486	42 877 574	126	no	4 586 081 693	3 689 490 402	3 661 753 542	59 908 909
SAC-018	43 871 234	41 680 457	126	no	4 513 315 129	3 373 461 120	3 338 768 179	83 803 779
SAC-019	44 377 864	42 156 351	126	no	4 565 324 199	3 488 622 173	3 468 402 076	53 698 238
SAC-020	44 140 320	41 055 107	126	no	4 540 910 980	3 368 594 025	3 314 498 883	54 952 587
SAC-021	44 250 378	41 557 897	126	no	4 552 212 117	3 362 792 674	3 329 970 831	62 281 855
SAC-022	49 058 224	46 191 194	126	no	5 046 727 279	3 792 658 586	3 762 515 431	67 669 951
SAC-023	40 738 346	38 197 979	126	no	4 190 895 100	3 262 355 990	3 239 157 072	42 063 870
SAC-024	46 084 922	43 531 206	126	no	4 740 835 720	3 407 693 802	3 391 838 301	62 487 745

Continued on the next page...

Table 1—continued

Sample ID	Original data	Without human DNA (noHum)	Avg. read length	Paired-end seq.	Total number of 24-mers	Number of unique 24-mers in the k-mer database, $ci = 1$	Number of unique 24-mers in the k-mer database (noHum), $ci = 1$	Number of unique 24-mers in the k-mer database (noHum), $ci = 4$
SAC-025	40 899 724	38 276 389	126	no	4 207 548 055	3 124 794 806	3 098 558 312	54 090 286
SAC-026	50 776 892	48 200 119	126	no	5 223 554 345	3 765 593 400	3 751 977 420	77 179 688
SAC-027	40 886 598	38 380 063	126	no	4 206 192 030	3 019 390 652	2 994 509 964	53 740 360
SAC-028	46 160 486	43 762 062	126	no	4 748 623 297	2 921 847 178	2 915 788 272	68 455 987
SAC-029	43 624 656	42 076 920	126	no	4 487 828 937	3 741 691 504	3 724 749 120	40 457 571
SAC-030	44 575 336	26 844 359	126	no	4 585 652 739	3 589 497 890	3 072 481 873	32 788 653
SAC-031	41 452 634	39 771 323	126	no	4 264 454 081	3 314 347 476	3 304 430 035	53 074 107
SAC-032	42 581 530	40 449 298	126	no	4 380 582 724	3 379 448 058	3 359 752 649	53 822 100
SAC-033	41 365 694	39 042 125	126	no	4 255 501 896	3 177 471 219	3 167 098 440	53 489 485
SAC-034	42 825 310	17 744 710	126	no	4 405 514 284	3 177 038 361	2 487 838 085	37 323 968
SCL-001	33 689 527	20 030 936	151	yes	8 618 533 043	4 392 358 848	3 805 650 023	286 989 740
SCL-002	30 750 895	25 053 675	151	yes	7 865 484 996	4 421 544 331	4 213 172 911	264 178 757
SCL-003	30 707 014	24 201 608	151	yes	7 855 646 107	3 757 740 715	3 590 584 822	237 418 176
SCL-004	30 317 867	19 643 679	151	yes	7 757 627 783	3 696 092 629	3 341 732 612	275 011 007
SCL-005	30 362 560	23 864 722	151	yes	7 767 932 294	4 123 735 793	3 907 889 948	275 601 675
SCL-006	30 372 515	22 395 452	151	yes	7 770 115 968	4 035 818 494	3 752 545 813	295 649 062
SCL-007	30 231 981	2 936 500	151	yes	7 733 731 167	3 400 892 506	2 247 159 636	209 279 142
SCL-008	30 038 721	27 043 032	151	yes	7 684 489 558	4 478 552 656	4 369 402 265	264 699 106
SCL-009	4 260 929	4 115 213	151	yes	1 090 743 257	205 680 900	204 542 457	45 790 381
SCL-010	3 428 431	1 887 391	151	yes	877 635 126	550 388 788	452 004 282	15 194 037
SCL-011	4 565 640	3 634 379	151	yes	1 168 741 053	706 234 451	671 060 251	16 009 689
SCL-012	3 476 735	2 841 074	151	yes	889 999 012	572 733 331	544 942 777	14 995 270
SCL-013	4 803 124	3 907 312	151	yes	1 229 537 129	606 465 309	598 274 527	11 692 250
SCL-014	4 730 548	3 779 747	151	yes	1 210 955 605	585 399 889	577 218 486	14 703 488
SCL-015	3 632 551	2 186 331	151	yes	929 884 299	531 858 974	453 093 894	13 923 932
SCL-016	3 172 389	2 791 247	151	yes	812 088 973	496 526 333	486 917 022	15 986 356
SCL-017	3 471 442	2 605 019	151	yes	888 642 982	454 748 869	431 421 617	9 982 461
SCL-018	6 162 164	4 565 824	151	yes	1 577 435 891	834 597 408	787 033 030	25 839 256
SCL-019	6 087 353	4 851 192	151	yes	1 558 281 580	773 150 163	758 308 350	24 535 166
SCL-020	3 648 818	3 298 504	151	yes	934 052 575	570 613 394	551 955 249	27 966 549
TOK-001	30 014 059	14 135 812	151	yes	7 681 193 251	3 235 791 279	2 603 468 821	386 637 932
TOK-002	29 949 377	17 100 317	151	yes	7 662 646 719	3 522 159 661	2 959 403 931	266 509 509
TOK-003	29 834 135	6 057 190	151	yes	7 631 610 738	3 544 385 878	2 520 743 670	210 138 709
TOK-004	29 845 743	7 872 293	151	yes	7 635 515 639	3 519 366 580	2 574 754 239	226 337 205

Continued on the next page...

Table 1—continued

Sample ID	Original data	Without human DNA (noHum)	Avg. read length	Paired-end seq.	Total number of 24-mers	Number of unique 24-mers in the *k*-mer database, $ci = 1$	Number of unique 24-mers in the *k*-mer database (noHum), $ci = 1$	Number of unique 24-mers in the *k*-mer database (noHum), $ci = 4$
TOK-005	29 916 014	21 714 115	151	yes	7 653 200 678	3 298 059 363	3 126 329 713	315 910 823
TOK-006	29 666 543	15 015 068	151	yes	7 589 815 497	3 528 315 586	2 895 851 631	263 813 314
TOK-007	29 872 086	12 590 700	151	yes	7 643 723 709	3 836 875 669	3 098 751 543	220 569 389
TOK-008	12 270 726	7 080 657	151	yes	3 139 140 722	1 484 321 236	1 308 077 091	100 140 785
TOK-009	29 687 454	11 883 669	151	yes	7 595 505 314	3 414 746 667	2 658 657 735	239 495 327
TOK-010	28 264 061	8 331 046	151	yes	7 230 943 849	3 592 195 704	2 727 712 415	203 032 576
TOK-011	29 636 642	10 135 691	151	yes	7 581 700 646	3 464 388 951	2 634 854 119	234 857 120
TOK-012	29 569 747	13 925 225	151	yes	7 564 078 534	3 378 913 875	2 763 704 224	273 302 832
TOK-013	29 749 452	15 600 617	151	yes	7 611 649 761	4 179 873 710	3 531 860 169	219 491 125
TOK-014	22 781 753	8 166 275	151	yes	5 828 232 151	2 784 770 651	2 135 404 375	171 577 403
TOK-015	27 645 194	8 435 794	151	yes	7 072 565 678	3 413 708 555	2 563 230 827	208 376 081
TOK-016	29 277 371	15 321 947	151	yes	7 490 200 084	4 145 673 700	3 492 204 761	223 033 255
TOK-017	29 681 515	5 446 380	151	yes	7 595 998 609	3 431 909 275	2 410 600 167	207 600 442
TOK-018	29 691 352	12 105 065	151	yes	7 597 459 858	3 650 184 995	2 886 246 950	247 402 407
TOK-019	28 576 785	12 063 919	151	yes	7 310 932 946	2 981 943 764	2 315 461 289	367 118 469
TOK-020	28 756 554	20 467 987	151	yes	7 356 956 090	3 120 645 590	2 834 100 161	314 277 363
